# Eco-evolutionary modelling of microbial syntrophy indicates the robustness of cross-feeding over cross-facilitation

**DOI:** 10.1038/s41598-023-27421-w

**Published:** 2023-01-17

**Authors:** G. Boza, G. Barabás, I. Scheuring, I. Zachar

**Affiliations:** 1 Institute of Evolution, MTA Centre for Ecological Research, Budapest, Hungary; 2 ASA Program, International Institute for Applied Systems Analysis (IIASA), Laxenburg, Austria; 3 Centre for Social Sciences, Budapest, Hungary; 4 Division of Ecological and Environmental Modeling, Linköping University, Linköping, Sweden; 5 Department of Plant Systematics, Ecology and Theoretical Biology, Eötvös Loránd University, Budapest, Hungary; 6 Parmenides Foundation, Centre for the Conceptual Foundation of Science, Pullach Im Isartal, Germany

**Keywords:** Evolutionary ecology, Microbial ecology, Evolution

## Abstract

Syntrophic cooperation among prokaryotes is ubiquitous and diverse. It relies on unilateral or mutual aid that may be both catalytic and metabolic in nature. Hypotheses of eukaryotic origins claim that mitochondrial endosymbiosis emerged from mutually beneficial syntrophy of archaeal and bacterial partners. However, there are no other examples of prokaryotic syntrophy leading to endosymbiosis. One potential reason is that when externalized products become public goods, they incite social conflict due to selfish mutants that may undermine any mutualistic interactions. To rigorously evaluate these arguments, here we construct a general mathematical framework of the ecology and evolution of different types of syntrophic partnerships. We do so both in a general microbial and in a eukaryogenetic context. Studying the case where partners cross-feed on each other’s self-inhibiting waste, we show that cooperative partnerships will eventually dominate over selfish mutants. By contrast, systems where producers actively secrete enzymes that cross-facilitate their partners’ resource consumption are not robust against cheaters over evolutionary time. We conclude that cross-facilitation is unlikely to provide an adequate syntrophic origin for endosymbiosis, but that cross-feeding mutualisms may indeed have played that role.

## Introduction

Microbial interactions include a wide range of mechanisms that shape not only the locally interacting pair but often the whole community or the larger ecosystem through externalized products^1,2^. Metabolite-based cooperation, syntrophy, is often crucial for the stable coexistence of microbial communities^3–5^. The term syntrophy (“co-feeding”) gradually increased in scope to cover a diverse range of both trophic and catalytic interactions, leading to unidirectional or mutual aid^6^ that, in general, allow a community to survive in environments where individuals cannot^7^. Differences in the specific mechanisms of syntrophy likely have fundamentally different consequences for the eco-evolutionary dynamics of species.

While syntrophy is ubiquitous in the prokaryotic domain (likely responsible for the unculturability of many prokaryotes^3,8,9^), partnerships stop at ectosymbioses, never achieving true endosymbiosis via physical integration^10^. It is intriguing that we do not find further examples of purely prokaryotic endosymbioses (i.e., not embedded in a eukaryotic overhost), other than the singular putative mitochondriogenetic origin^11^. While there are many analogies to the endosymbiotic origin of mitochondria, rendering eukaryogenesis a perhaps less unique major evolutionary transition^12^, it is perfectly valid and relevant to ask why prokaryotic syntrophy has not lead to magnitudes more endosymbiotic integrations over ~ 4 billion years (that we know of). After all, multicellularity has evolved multiple times, independently. Besides metabolic compatibility and adaptive superiority, the process likely required a long and stable period during which species could coevolve without interruption from third parties. It is unknown whether and which types of syntrophy can be stably maintained for a prolonged time, withstanding the inevitable invasion of cheaters and other biotic and abiotic disturbances, especially regarding eukaryogenetic scenarios. We set out to evaluate the ecological and evolutionary robustness of different syntrophic mechanisms via mathematical modelling. Investigations may not only provide insight into prokaryotic integration (or the apparent lack of it) but could improve our understanding of the singularity of eukaryogenesis.

**Figure 1:**
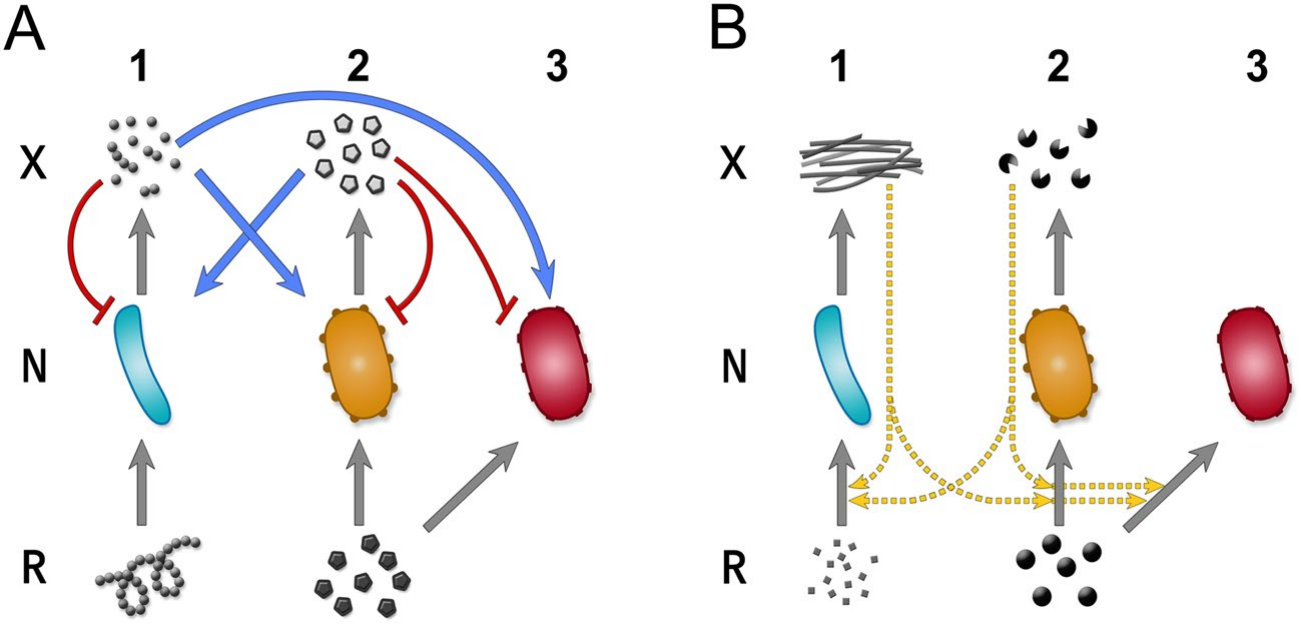
Models of cross-feeding (**A**) and cross-facilitation (**B**). Species $N_i$ consumes resource $R_i$ and produces $X_i$ (grey arrows). (**A**) Trophic cross-feeding. Product $X_i$ represents a self-inhibiting (red curve) waste material of species $N_i$ that can be consumed by species $N_j$, serving directly as food (blue arrows). (**B**) Enzymatic cross-facilitation or collaborative-feeding. Product $X_i$ of species $N_i$ is an enzyme providing cooperative help (e.g. extracellular digestion of resources, formally equivalent to any form of indirect aid of a protective mutualism) to partner species and to itself by enhancing the consumption of resources $R_i$ (dashed yellow arrows). In both cases, a mutant species $N_3$ (red cell) may appear that inherits the properties and interactions of $N_2$ (yellow cell) but does not produce anything.

The broadest definition of syntrophy covers all metabolic cooperation that positively affects the population growth of another species^13^. Formally, cooperation is equivalent to cross-catalysed replication of molecules in chemical systems not involving cells, e.g. ribozymes catalysing the replication of other ribozymes. Between (cellular) organisms cooperation has the same second-order kinetics as cross-catalysis between chemical replicators^14^, without the fast association-dissociation dynamics of true chemical catalysts, as in this case, species aid each other via externalized molecules. These may directly serve as nutrients for the partner (e.g. living on the byproduct of another species^13,15^; called here *cross-feeding* in the narrow sense Fig. 1A) or they may facilitate each other indirectly by e.g. enabling resources (via. e.g. digestive enzymes^5^; called collaborative feeding, or more generally *cross-facilitation* Fig. 1B). Collaborative-feeding occurs when two distinct lineages secrete the same extracellular enzyme, a public good, and together increase the specific activity of that enzyme, rendering an otherwise inaccessible resource accessible, facilitating both partners' growth^5^, or via the benefits delivered by another form of facilitation, transportation, or protection mutualisms^16–18^. Microbial interaction models often focus solely on the phenomenological level of species and fail to capture the fundamentally distinct nature of the different mechanisms involved^19^. Note that, while cross-fed metabolites or external enzymes may convey a cooperative benefit, neither catalyses the reproduction of partners *directly*. The difference lies in their inhibitory effect, production cost, and reusability.

Most microorganisms are found to be auxotrophic, lacking essential pathways, depending on extracellular sources of amino acids, vitamins, and cofactors^20^, implying nutritional cross-feeding^3^. A diversity of externalized molecules may transmit cooperative effects, e.g., digestive enzymes^4,5,21–23^, signals^24^, protective matrix materials^25^, siderophores^26,27^, metallophores, biosurfactants^28^, antibiotics^29^, amino acids, vitamins, and other cofactors (for a review, see^5^). The common feature of exchanged products is the private or collective benefit they exert, acting as private, semi-private, or common goods^30,31^. Metabolic benefits are harnessed either by directly consuming produced metabolites (e.g., nutritional mutualism, waste consumption^13^), or via the catalytic effects of products that remain reusable (e.g. extracellular enzymes^5^). For further examples, see Supplementary Table S1.

A particularly important type of nutritional cross-feeding is the detoxification of inhibitory molecules, such as waste^13^ or reactive oxygen species^20^. Waste can accumulate in prohibitive concentrations internally, therefore disposing of it is beneficial for the producer (low cost). A partner that consumes the waste can effectively alleviate the external inhibitory effect on the producer, facilitating its growth. Mutual byproduct-consumption therefore may lead to reciprocal cooperation, or mutualism^32^. A textbook example is that of methanogenic syntrophies: the bacteria ferment lactate, producing hydrogen that inhibits the above process unless the archaeal partner consumes it in order to reduce carbon dioxide to methane^33^.

A fundamentally different mechanisms of syntrophic cooperation is mediated by products that provide indirect benefit as are not consumed by partners (hence we call it cross-facilitation to differentiate it from nutritional cross-feeding and to use a general term used in ecology for positive interactions^34^). Species can produce reusable catalytic factors that benefit not only the producer but the whole community. Extracellular enzymes that degrade complex substrates to forms that can be picked up by the producer and its neighbours^4^ can improve resource consumptions^5,23,35^. For large molecules, the cost of production and excretion can be substantial, especially compared to waste disposal. Protection mutualisms have similar effects, where factors produced by community members serve as non-consumable common goods (protective matrix of a biofilm^24,25^, bacteriocins serving as growth-inhibiting antimicrobial compounds^24^, or extracellular detoxification and the neutralization of inhibitory substances, e.g., antibiotics^17,36^). Different (potentially prokaryotic) species may combine their extracellular enzymatic activities cooperatively^37^ to achieve enhanced growth and enable new niches^38^. Hybrid examples, with both cross-feeding and cross-facilitation, also exist^16^, e.g. in biofilms^24^. For a list of various examples, see Supplementary Information SI 1.

Public goods may benefit species but they also generate social conflict and attract cheaters that do not invest into production while benefit from the goods^23,39^. Numerous experiments have demonstrated that mutant strains or emerging ecotypes can stably coexist within the community (clonal cooperation^1^) due to e.g. differential use of resources^40^ and cross-feeding^41^, especially when such division of metabolic labour is engineered^42,43^, often demonstrating higher fitness or productivity^3,41,44,45^. However, such compatibility between strains represents a best-case scenario. Selfish mutants not contributing anything but competing more effectively for resources are more likely to appear^46^, and they may ultimately win over strains that invest to a costly cooperative act^47^. Emergent cheater strategies have been observed in various experimental systems, including biofilm formation by *P. aeruginosa* where biofilm thickness and health was reduced by non-contributing strains^48^ or the survival of whole biofilm was sabotaged by such defecting types in *P. fluorescens*^49^. Non-producers exploiting public resources can turn saved production costs to higher growth rates and may outcompete producers^35^, leading to the ‘tragedy of the commons’^50^ and the collapse of the community^24,51^. Consequently, all forms of syntrophy are prone to be disrupted by cheater mutants that reduce their costs at the expense of producers.

Despite the obvious differences between cross-feeding and cross-facilitation, it is not trivial which can lead to evolutionarily stable mutualism that could (in principle) account for the (endo)symbiotic integration of prokaryotic partners. We ask whether and which syntrophy can be stable against free-riders. Here we provide a mathematical model to investigate the ecological and evolutionary dynamics and robustness of symmetric and asymmetric cross-feeding and cross-facilitation of two unspecified microbial species in syntrophic interaction, potentially with mutants to appear (Fig. 1). Based on our analysis, cross-facilitation appears to be an unlikely (but not impossible) candidate for serving as the syntrophic origin for stable partnership or endosymbiosis. Cross-feeding mutualisms, however, may indeed have played that role.

## Results

We have modelled a theoretical partnership of unrelated microbial species 1 and 2 that belong to different guilds with different metabolic needs and therefore are limited by two independent resources ${R}_{1}$ and ${R}_{2}$, hence their coexistence is guaranteed. They secrete specific metabolites ${X}_{1}$ and ${X}_{2}$ to the environment (interpreted either as energy-rich waste or as enzymes), which benefit themselves and possibly the other species (Fig. 1). We assume linear consumer-resource dynamics with fast resources^52^. That is, resource uptake is fast compared to cellular growth, so resource concentrations are considered to be in steady state on the time of scale of the consumers’ dynamics. We also assume a well-mixed environment where externalized products are immediately available to anyone. Therefore, any product is potentially a publicly available good. In case of cross-feeding, the secreted metabolite is waste that may self-inhibit (Fig. 1A). In collaborative-feeding (a case of cross-facilitation), species secrete enzymes that catalyse reactions in the environment, improving resource consumption for themselves and other species (Fig. 1B). A cheater mutant species 3 (technically an ecotype instead of a bona fide new biological species), can potentially emerge that lives on the same resource as its parent but may invest less, or even nothing, in production to increase its survival rate (or invest more into production at the expense of survival). While cooperative strains may also emerge^1^, we deliberately chose a worst-case scenario to test the stability of partnerships under worst conditions.

**Figure 2:**
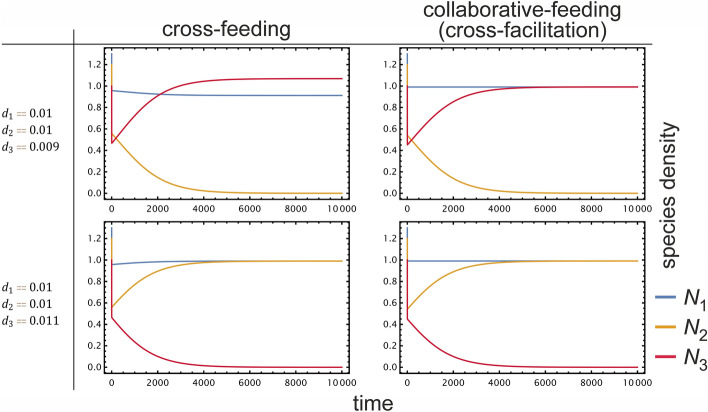
Time-evolution of tripartite microbial systems with cross-feeding or collaborative-feeding interactions, depending on interaction type and per-capita mortality rate ($d$). The *x*-axis represents time, the *y*-axis represents species density. In both interaction types, only two species can stably coexist, the third one being extinct in the equilibrium. Whenever the mutant species (red) has smaller mortality than its parent ($d_{3}<d_{2}$, top row), it can invade, causing the extinction of the resident parent (yellow). If the mutant mortality is larger (bottom row), it cannot invade. Parameters are $\left\{b_{1}=b_{2}=b_{3}=1,r_{1}=r_{2}=1,{\delta }_{1}={\delta }_{2}=0.1,k_{1}=k_{2}=k_{3}=0.1,c_{1}=c_{2}=c_{3}=1,d_{1}=d_{2}=0.01,m_{1}=m_{2}=m_{3}=1,s_{11}=s_{12}=s_{13}=s_{21}=s_{22}=s_{23}=s_{31}=s_{32}=s_{33}=0.1,g_{1}=g_{2}=g_{3} =1,h_{1}=h_{2}=h_{3}=1,w_{1}=w_{2}=w_{3}=1\right\}$; parameters are explained in Table S2.

Based on these characteristics of the system, we have built a family of simple mathematical models to assess which types of syntrophy and interaction network topology are more likely to yield evolutionary stable, pairwise symbiosis. These serve as the first formal models of syntrophy that explicitly test a crucial aspect of metabolic interactions considered to have been relevant at the onset of eukaryogenesis. See Methods for the mathematical details, Fig. 2 for some typical time series produced by the models, and Table S2 for parameters.

### Cross-feeding

#### Coexistence of cross feeders

First, we examine byproduct cross-feeding [Fig. 1A, Eq. (4)]. Byproduct metabolites often accumulate internally, stalling the metabolism of the organism. Hence disposing them is beneficial, while retaining them internally is costly^13,53^. In the simplest case, we omit the potential of disposed waste for self-inhibition (${h}_{i}=0$ for all species $i$), to make analytical investigations simpler. Without metabolic cross-feeding (${g}_{i}=0$ for all $i$), species 1 reaches its carrying capacity independently of the other species, while species 2 and 3 become complete competitors for resource ${R}_{2}$. As a result, the competitor which utilises the resource more effectively by having a lower ${b}_{i}/{d}_{i}$ value will exclude the other (${R}^{*}$-rule^54^).

Cross-feeding couples the dynamics of the species, rendering the analysis more complex. When self-inhibition is negligible (${h}_{1}={h}_{2}=0$) but species cross-feed (${g}_{1},{g}_{2}>0$), we show that species 1 and 2 are in stable coexistence when their net growth rates are sufficiently high; otherwise both species go extinct (Supplementary Information SI 3). Assuming that species can grow independently of each other (i.e., cross-feeding is facultative) and under biologically realistic conditions, the dynamics always lead to stable coexistence.

Next, we include self-inhibition by the the waste metabolites. While secretion can effectively dispose waste, it can still accumulate externally, potentially causing self-inhibition. For instance, hydrogen-producing bacteria cannot grow due to the inhibiting effect of accumulating hydrogen when it is not consumed by methanogenic partners^13^. With self-inhibition taken into account (${h}_{1},{h}_{2}>0$), the dynamics of species 1 and 2 remain qualitatively unchanged despite the more complex dynamical equations. Following the analysis of the simpler case without self-inhibition, we conjecture that a single, globally stable internal fixed point always exists, assuming realistic parameter combinations and positive growth without the partner (SI 5). Extensive numerical simulations indicate that this is indeed the case, and that species concentrations tend to a globally stable internal fixed point if net growth rates are sufficiently high (SI 4).

These results indicate that once a pair of species have established mutual cross-feeding, they remain in stable coexistence against (small) perturbations in density or parameter values. Next, we examine how stable a partnership is against cheating mutants that may not produce compounds benefiting or inhibiting anyone.

#### Invasion of mutants

Here, we investigate whether a mutant species can invade the community in equilibrium. We assume that the mutant (species 3, a rare mutant of species 2) is unable to efficiently dispose of its waste product, hence it must pay a cost, compared to species 1 and 2 that excrete waste metabolites (Fig. 1A). Accordingly, the mortality rate of species 3 is larger than that of species 2 (${d}_{3}>{d}_{2}$). Because of the symmetry of the model, it is indifferent which resident species (1 or 2) generates the mutant.

We map out when the mutant species 3 can invade the partnership of species 1 and 2 being in stable coexistence. We also examine the case of species 2 being the invading mutant of species 3, with species 3 being the stable partner of 1 (SI 6). Depending on model parameters, we determine the direction of evolution (i.e., species 3 exchanges species 2 because species 3 can invade while species 2 cannot or vice versa and the case when species 2 and 3 coexist because of mutual invasion). To make the analysis tractable, we again assume no self-inhibition (${h}_{1}={h}_{2}=0$).

Apart from assuming that withholding $${X}_{2}$$ increases the death rate of species 3 compared to species 2 ($${d}_{3}>{d}_{2})$$, we assume that the conversion efficiencies of resources and byproducts remain the same ($${{b}_{2}={b}_{3}, g}_{2}={g}_{3})$$ for the mutant. In this case species 3 cannot invade the pair of species 1 and 2, while species 2 invades successfully the pair of species 1 and 3. That is, species 3 cannot coexist with species 2 and the species (1, 2) subsystem is resistant against the invasion of selfish mutants (SI 6). It is natural to assume that increasing the rate of resource uptake $$g$$ correlates with higher per-capita death rates $$d$$, so these two variables are in positive trade-off (there are plenty of examples for such trade-offs between microbial traits^55,56^). Accordingly, in case the selfish mutant 3’ utilizes the byproduct more efficiently than the resident ($${g}_{3}^{^{\prime}}>{g}_{2})$$, it must pay an even larger cost, realized in an even higher mortality rate ($${d}_{3}^{^{\prime}}>{d}_{3}>{d}_{2})$$. Depending on the trade-off between mortality rate $$d$$ and conversion efficiency $$g$$, one of the species can exclude the other or they can mutually invade each other, thus all three species can coexist (SI 6). The evolution of these correlated traits is studied in more detail using adaptive dynamics in the next section.

**Figure 3:**
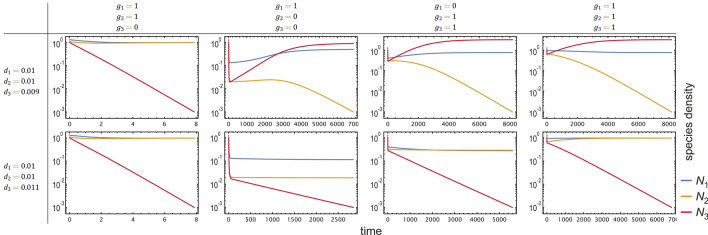
Time evolution of a cross-feeding partnership with waste product inhibition with a mutant species appearing (red), depending on the directionality of cross-feeding ($g$) and mortality ($d$). We assume that species 3 (a mutant of species 2, yellow) inherits the resource utilization of species 2 ($g_{3}=g_{2}$, except in the first column). The *x*-axis represents time, the *y*-axis represents density on a logarithmic scale. Species 1 and 2 cause the mutant species 3 to go extinct whenever $d_{2}<d_{3}$, even if there is no cross-feeding at all. That is, the apparent survival of producers against cheaters is not because of cross-feeding but because of the higher assumed mortality for cheaters. When there is cross-feeding between species 1 and 2 ($g_{1}=g_{2}=1,g_{3}=0$), coexistence of species 1 and 2 is independent of the ratio of $d_{1,2}/d_{3}$. That is, species 3 has no chance of persisting, even if it has much smaller mortality than the others. When there is asymmetric cross-feeding such that species 2,3 cannot feed on $X_{1}$ ($g_{1}=1,g_{2}=g_{3}=0$), or species 1 cannot feed on $X_{2}$ ($g_{1}=0,g_{2}=g_{3}=1$), or everyone can cross-feed ($g_{1}=g_{2}=g_{3}=1$), species 3 can replace species 2 only if it has smaller death rate $d_{3}<d_{2}$. Results are qualitatively the same when inhibition is omitted ($h_{1}=h_{2}=h_{3}=0$). Parameters are $\left\{r_{1}=r_{2}=1,c_{1}=c_{2}=c_{3}=1,b_{1}=1,b_{2}=b_{3}=0.1,d_{1}=d_{2}=0.01,{\delta }_{1}={\delta }_{2}=0.1,k_{1}=k_{2}=1,h_{1}=h_{2}=h_{3}=1,w_{1}=w_{2}=w_{3}=1\right\}$.

Adding self-inhibition of waste (${h}_{1},{h}_{2}>0$), we observe the same qualitative behaviour via numerical simulations. See Fig. 3 for the characteristic time-evolution of the various cases. If the subsystem of species (1, 2) has a stable internal fixed point (a theorem for ${h}_{1},{h}_{2}=0$ and a conjecture otherwise which nevertheless seems to be the case), then one can prove for ${h}_{1},{h}_{2}>0,{h}_{2}={h}_{3}$ that species 3 cannot invade if ${d}_{3}>{d}_{2}$. At the same time, species 2 can invade the species (1, 3) subsystem (SI 6).

#### Adaptive dynamics

To simulate the evolution of cross-feeding, we implemented a numerical version of adaptive dynamics^57^ for our model. We assume that consumption efficiency $g=g(z)$ and mortality rate $d=d\left(z\right)$ both depending on an underlying trait $z$, and are in trade-off. Assuming that the trait is determined by many genes, we expect mutations incur only small actual changes in trait value. The trade-off between $g$ and $d$, governed by equations [Eq. (7)], ensures that a higher byproduct consumption rate can only be attained at the cost of increased mortality. In our adaptive dynamics model, we check whether mutants that only slightly differ in their trait value from a resident species can invade and replace the resident or not (for details, see SI 7, 8).

**Figure 4:**
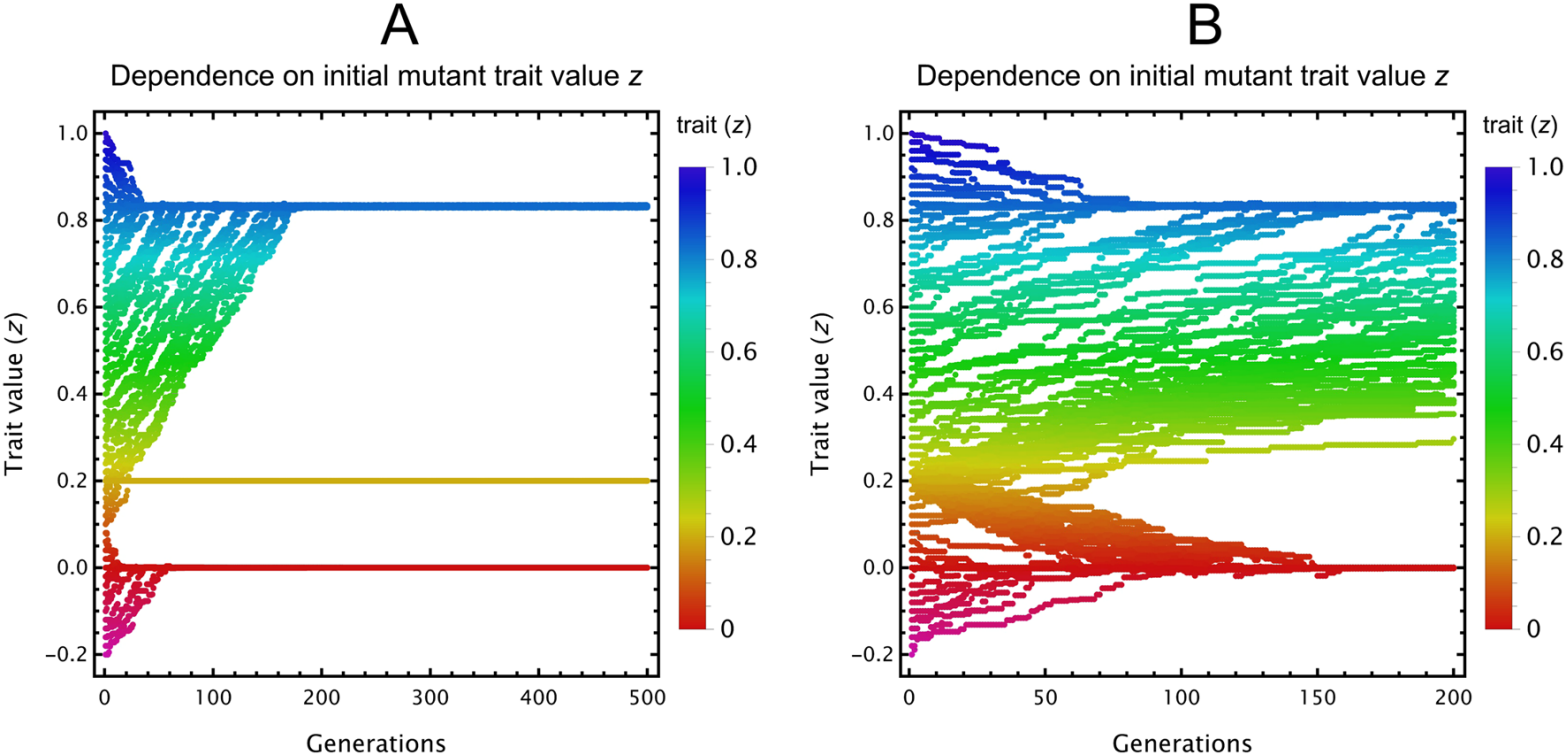
Evolutionary trajectories of resident and mutant cross-feeding species with inhibition, throughout successive generations of invasions in case only one (**A**) or both species can mutate (**B**). Adaptive dynamics simulations start from different initial mutant trait values ($z$). Trait value (y-axis) is shown against generations (x-axis) for both mutant classes. Colours correspond to trait value, opacity to the relative equilibrium density of the various species present in the actual population. (**A**) Only species 2 can mutate, species 1 is fixed (orange line at $z_{1}=0.2$). Evolutionary trajectories of species 2 converge to either the equilibrium trait value at around $z\approx 0.82$ (in case the starting trait is larger than about 0.1), or to the one at $z=0$. (**B**) Both species may evolve. To achieve mutually positive trait values (implying cross-feeding), species 1 must have a starting trait over 0.2. Trajectories starting from around this critical $z\approx 0.2$ may end up at either equilibrium due to stochasticity. When both species have positive equilibrium trait values, mutual cross-feeding evolves. In case one (or both) species converge to $z=0$, there is no cross-feeding as the trait value defines low mortality with negligible cross-feeding efficiency. Parameters are $\left\{t_{inv}={10}^{4},n_{inv}={10}^{-2},{\mu }_{SD}={10}^{-2},n_{\theta }={10}^{-3},G=300,z_{1}=0.2,b_{1}=1,b_{2}=0.1,c_{1}=1,c_{2}=0.1,k_{1}=1,k_{2}=0.1,r_{1}=r_{2}=1,{\delta }_{1}={\delta }_{2}=0.1,w_{1}=w_{2}=1,h_{1}=h_{2}=1,\overline{z}=0.5,\sigma =0.2,\eta =0.1\right\}$.

When only species 2 evolves without inhibition (SI 7), we observe that species 2 experiences directional selection towards a trait value $z$ that provides the best compromise between getting a benefit from cross-feeding without having an excessively large mortality rate. This evolutionary state of mutual cross-feeding is both locally and globally stable against invasion of other mutant, even against potential cheaters whose trait values are not close to that of species 2 at the end. We checked what happens when both species evolve, and when inhibition of waste is imposed (SI 8), to arrive at results qualitatively the same (Fig. 4).

### Cross-facilitation

#### Coexistence of collaborative feeders

Next, we examine collaborative-feeding, a specific case of cross-facilitation [Fig. 1B, Eq. (6)]. We assume that the extracellular metabolic product ${X}_{i}$ is an enzyme that has evolved to improve the producer’s resource consumption^21,23^. Consequently, producing and secreting this molecule is costly. On the other hand, the enzyme, when externalized, benefits not only the producer but potentially everyone else in the vicinity, improving their resource consumption efficiency.

#### Invasion of mutants

The subsystem of species 1 and 2 persists only if ${r}_{i}{b}_{i}-{d}_{i}>0$ for both (see SI 9, 11 for analytical considerations and SI 10 for numerical support). The mutant species 3 does not produce the enzyme (${k}_{3}=0$), hence it does not pay any production or secretion cost: ${{r}_{2}b}_{2}-{d}_{2}>{{r}_{2}b}_{3}-{d}_{3}$. As a result, species 3 does not produce a public good but still benefits from ${X}_{1}$. If the mutant does not invest into production, it generally wins over species 2, a realization of the tragedy of the commons^50^. It is easy to show that if enzymatic efficiency for species 2 is (roughly) identical to that of species 3 (${s}_{22}={s}_{32}$, ${s}_{21}={s}_{31}$), then species 3 always excludes species 2 because its total growth rate is always larger than that of species 2 ($\frac{1}{{n}_{3}}\frac{d{n}_{3}}{dt}>\frac{1}{{n}_{2}}\frac{d{n}_{2}}{dt}$ for every ${n}_{1},{n}_{2}>0$).

We note here that if there is some exclusively private benefit of producing an enzyme (or not producing it has an increased cost, like in case of waste), then the above simple selection dynamics no longer holds. However, since we assume a well-mixed system, such private benefits can be ignored. After species 2 has gone extinct, the dynamics of species 1 becomes independent of species 3. Thus, species 1 reaches its equilibrium concentration $${\widehat{n}}_{1}>0$$ if its net growth rate is positive ($${r}_{1}{b}_{1}-{d}_{1}>0)$$. Consequently, after species 1 reaches equilibrium, the dynamics of species 3 will depend only on its concentration, which leads to a positive equilibrium concentration too if $${r}_{2}{b}_{3}-{d}_{3}>0$$. This effectively means that *mutual catalytic aid is not stable* against the invasion of cheaters. After the invasion of the selfish species 3, species 1 coexists with the selfish invader. Imagine now that a new mutant of species 1 (species 1’) emerges, which does not produce enzyme 1 but utilizes it as effectively as species 1. Using the same argument as above, species 1’ will outcompete species 1. Consequently, the reciprocal catalytic help disappears ultimately.

#### Adaptive dynamics

**Figure 5:**
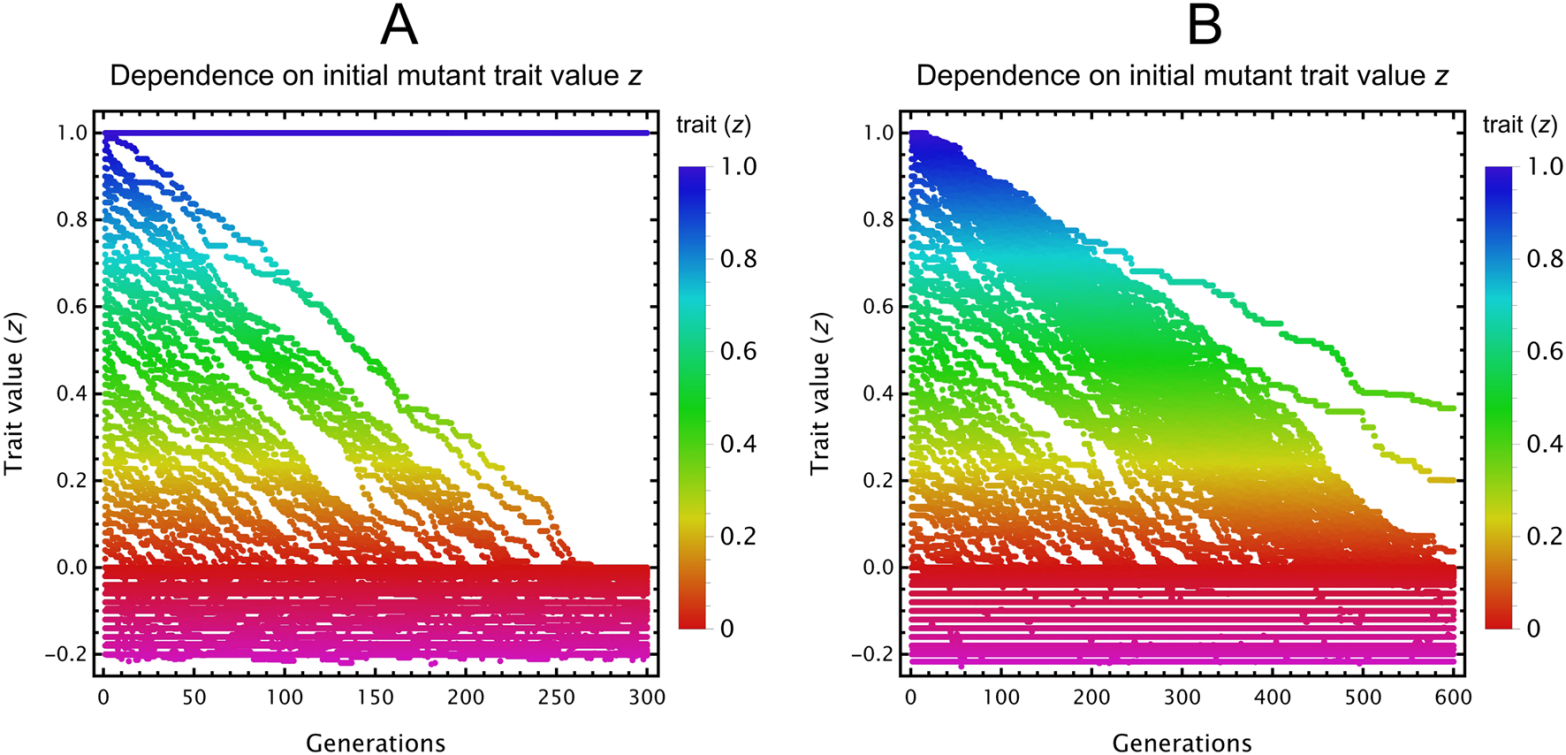
Adaptive dynamics of cross-facilitating species. (**A**) Only species 2 can evolve, species 1 is fixed (blue line at $z_{1}=1$). (**B**) Both species may evolve. In both cases, species evolve toward $z\approx 0$, where mortality is $d\approx 0$ and the production rate $k$ of the common good is also $\approx 0$. Parameters: $\left\{t_{inv}=5 \cdot {10}^{5},n_{inv}={10}^{-2},{\mu }_{SD}={10}^{-2},n_{\theta }={10}^{-2},G=600,z_{1}=1,b_{1}=1,b_{2}=0.1,c_{1}=1,c_{2}=0.1,r_{1}=r_{2}=1,{\delta }_{1}={\delta }_{2}=0.1,m_{1}=m_{2}=1,s_{11}=s_{12}=s_{21}=s_{22}=0.1,\overline{z}=0.5,\sigma =0.2,\eta =0.1\right\}$. Species at or below $z=0$ have $d=0$; they therefore become effectively identical copies of each other, leading to their neutral coexistence. For further details, see Fig. 4.

Next, we apply the same procedure of adaptive dynamics to cross-facilitation as we have done to cross-feeders. Since ${X}_{i}$ is an enzyme, it is beneficial to anyone that can access it, while producing it is costly. We therefore assume a trade-off between mortality $d\left(z\right)$ and production rate $k\left(z\right)$, common among microbes^58^, which ensures a larger cost paid in relative growth when more enzyme is produced per unit time [SI 12, Eq. (S9)]. Results indicate that cross-facilitation cooperation gets disrupted whenever there is a potential for cheaters to appear (Fig. 5).

We also investigate the cross-facilitation situation in which enzymes are species-specific with different functions, facilitating the digestion of a single resource only, while making it available for consumption for all species. This leads to qualitatively similar results (Fig. S15 in SI 13), supporting our claim that it is the costly nature of (enzyme) production that leads to the ultimate demise of cooperation in metabolic communities. The same holds for any hybrid case, where one species is cross-feeding the other with waste, while the other species produces an enzyme that benefits both (Fig. 7(4)): cross-facilitation is lost at the evolutionary equilibrium (SI 14 and Fig. S17).

## Discussion

Microbial communities are widespread in almost every habitat on Earth. Interactions among species are dominantly mediated by externalized metabolites^5^. Facultative syntrophies and auxotrophies are both common, where partners facultatively or obligately depend on products of others^6^. The latter may be the potential cause of the unculturability of prokaryotes^3,8,59^. The ubiquity of metabolic cooperation among microbes indicates that partnerships of complementary metabolisms can easily form, even without prior coevolutionary history^60^.

However, genome-scale metabolic networks indicate that despite the likelihood of metabolic compatibility among species, it is insufficient to compensate for the increased costs associated with satisfying two biomass requirements instead of just one, leading to reduced growth of the pair against free-living competitors^61^. Metabolically cooperating bacteria (even obligately dependent ones) can regain autonomous metabolisms which disrupts cooperation^62^. When obligate dependence evolves, species can only survive if their partners survive too. Thus, partner-dependent strains are generally more prone to the ecological and evolutionary changes affecting their symbionts than autonomous lineages not depending (or not obligatorily) on partners^62,63^. It is likely that exclusive partnership can only evolve if the environment is stable enough to allow prolonged cooperative coupling and dependence without biotic or abiotic disturbances.

A recent comparative analysis suggests that mutualistic interactions are rare in natural microbial communities^2^ (but see^18^), except in highly stressful but stable environments, where the common stress factor forces species to collaborate^16^. Modelled communities having more auxotrophic strains were less robust to ecological disturbance^63^. On the other hand, mutualism-dominated communities may occupy more diverse niches and are more resilient to abiotic perturbations (e.g. nutrient changes) while being more susceptible to invasion as opposed to competitive communities^64^. Experiments have demonstrated that multi-member communities with complex interaction topologies tend to reduce to a few core species, and removing a keystone species further reduces the community to a single pair^65^.

Maintaining partnership of prokaryotes dependent on externalized metabolites is especially challenging as there are no sophisticated mechanisms for partner recognition and partner-specific close-contact^10^. Partners can be exchanged by functionally equivalent ones without detrimental effects, as recent in vitro experiments demonstrate for archaea^59^ and microbial communities in general^66^. Such dependence on the partner’s functional profile but tolerance against taxonomic change is likely common to all prokaryotes. These factors may explain why endosymbiosis is virtually unknown among free-living prokaryotes despite the ubiquity of syntrophy^10^ (prokaryotic endosymbioses exist^67^, but provide limited analogy to eukaryotic origin^10^). The singular putative example is the origin of eukaryotes and mitochondria^59,68,69^.

According to syntrophic hypotheses of mitochondrial origins, endosymbiosis emerged from the mutually beneficial, metabolite-mediated syntrophy of prokaryotic partners^69–71^ (also see^72^). Reconstructed metabolisms of ancient partners may even support their presumed early cooperation^68^. These hypotheses, in general, assume different ancestral metabolisms for host and symbiont, and that they belong to different domains. Asgards, close to the eukaryotic branch, are metabolically versatile and have the ability to grow lithoautotrophically, producing H_2_ from amino-acid degradation^59,68,69,73^. Alternative, mitochondria-late hypotheses assume that the interaction started out as physical and exploitative, where mutualism did not play a critical initial role and was established only later, if at all^74,75^.

**Figure 6:**
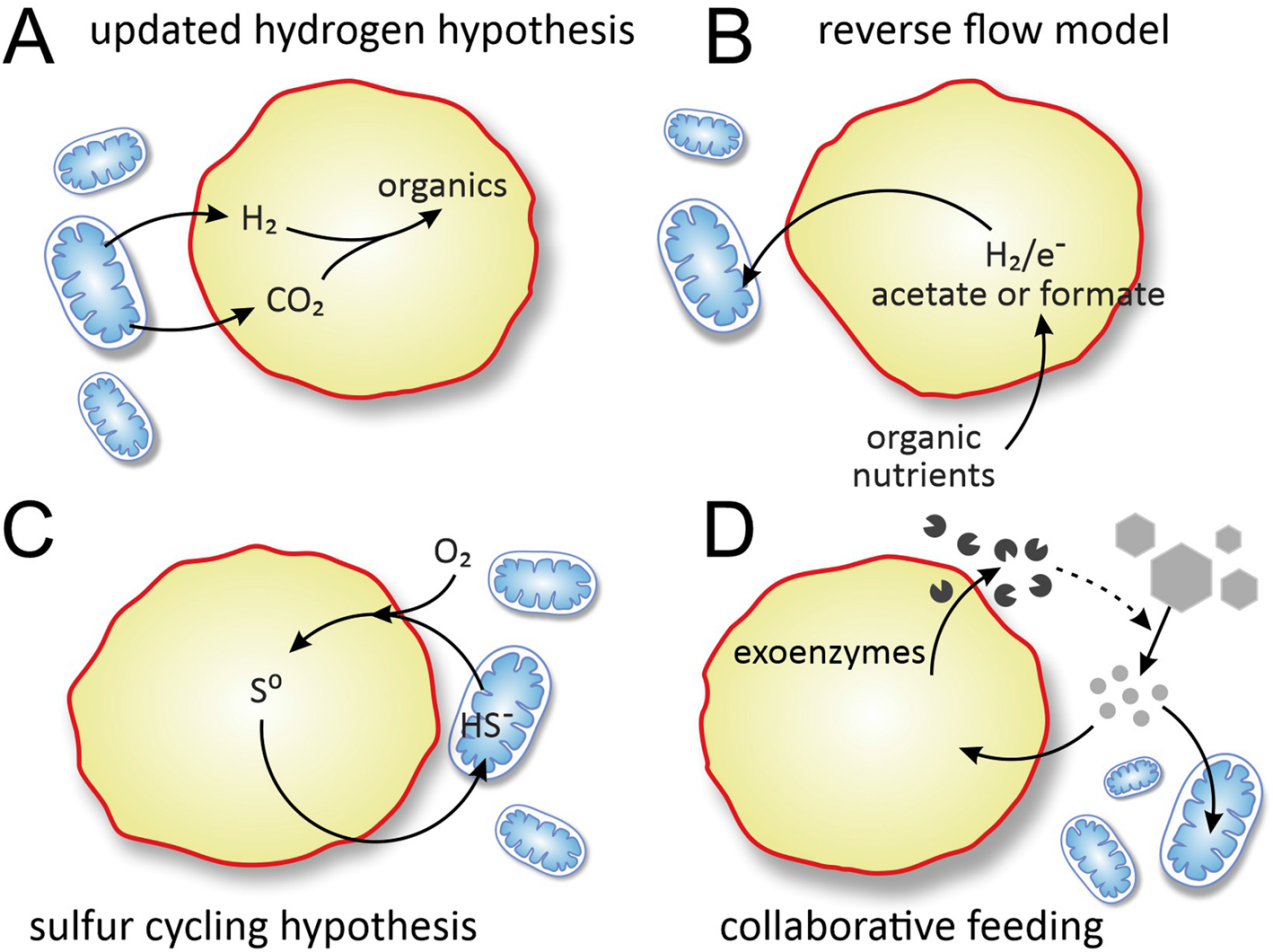
Comparison of various syntrophic eukaryogenetic hypotheses. The updated hydrogen hypothesis^68^ (**A**) and the reverse flow hypothesis^69^ (**B**) are examples of unidirectional (flow-through) byproduct consumption. The sulphur cycling hypothesis^78^ (**C**) is an example of symmetric recycle-type cross-feeding. (**D**) The collaborative-feeding model envisages a partner that secretes external enzymes that catalytically benefit both itself and any partner through making resources available to feed on (a case of cross-facilitation). The image is based on the image of^71^ (where a third partner is postulated, ignored in our model).

Syntrophic hypotheses assume product/waste syntrophy between the ancestral host and symbiont, where product(s) of one partner are directly metabolized by the other partner (Fig. 1A). A common assumption is that partners have exchanged hydrogen in one or the other direction (hydrogen hypothesis^68,70^ Fig. 6A, reverse flow hypothesis^69^ Fig. 6B). According to the latter, the ancestral host may have generated reducing equivalents utilized by the bacterial partner in the form of hydrogen, small reduced inorganic or organic compounds, or by direct electron transfer^69^. It is in the producers interest to dispose its waste, as otherwise it could accumulate to inhibitory amounts^13,53,76^. If this waste is consumed by a partner, both species can benefit, jointly performing a reaction that would otherwise be thermodynamically unfavourable for any one of them separately^72^. While the consumer’s act of feeding benefits the producer, this benefit is not because of reciprocal exchange of metabolites. In this asymmetrical setup (called *flow-through syntrophy*^76^), material flows in one direction and producers can unilaterally control consumers further down the chain. While a methanogenic host for mitochondria has been ruled out, methanogenic archaea are fitting examples of flow-through cross-feeding, as they are responsible for the efficient removal of hydrogen and formate produced by primary fermenters in the absence of other terminal electron acceptors^13,77^.

Syntrophic interactions can also be symmetric (reciprocal), when both species pass on metabolites to the other that convey the benefit. Sulphur-cycling via oxidation and reduction between *Sulphurospirillum* and *Chlorobium* enables a rapid exchange which rate depends on the (small) amount of sulphur (and the rate of its regeneration) that has a catalytic effect on both species (called *recycle syntrophy*^76^). According to the sulphur-cycling hypothesis (Fig. 6C), ancestral eukaryogenetic partners cycled sulphur repeatedly, in effect serving as an electron carrier between the two organisms^78^ (or in a tripartite endosymbiosis^71^). While modern phylogenomic results do not support mitochondria deriving from Rhodospirillaceae, it serves as an example of symmetric cross-feeding in contrast to unidirectional syntrophies.

Alternatively, one may assume that the initial interaction between partners was not direct feeding on leaked metabolites but mediated by secretions that provided indirect benefit for both parties by cross-facilitation (Fig. 1B). Most prokaryotic species live in surface-adhered, multispecies biofilms^79^ where all forms of syntrophies, nutritional and catalytic, are common^24^. For example, a catabolic exoenzyme of one species makes a resource available for everyone, e.g. cellulose^23,35^ (Fig. 6D). This catalytic help is formally equivalent to the effect of a product that provides protection for everyone and remains in the vicinity, e.g. (biofilm matrix^24,25^, antibiotics^29^ or antibiotic degrading enzymes^24^). We can envisage an early mitochondriogenetic partnership that benefited from a protective environment generated by a partner. However, whether the ancestral host and mitochondria co-evolved in a surface-bound community or as free living is presently unknown.

While syntrophic hypotheses gained considerable support due to the improved characterization of closest relative Asgard archaea^68,69,73,80–82^, modelling the initial interaction is grossly neglected. Assumed chemical compatibility and the potential for cooperation do not necessarily entail stable (ecological) coexistence of species, much less long term (evolutionary) stability against cheater mutants (cf.^61^). For endosymbiosis, especially prokaryotic, without phagocytosis or a nucleus, long term stable coevolution is necessary. Without modelling the early evolutionary ecology of these systems, evolutionary claims inevitably become superficial (as we have already argued^11^).

In this paper, we have designed a set of formal mathematical models of microbial interactions to compare the ecological and evolutionary stability and potential of different syntrophies. Our models are not particular to the prokaryotic domain and species may represent any unicellular organism. We have analysed which interaction type (nutritional cross-feeding or collaborative-feeding syntrophy) and interaction network topology (mutual or unilateral) is more robust against cheaters. We have examined from which type could evolution traverse to other types and which one can enable exclusively pairwise stable mutualism—the cornerstone of endosymbiosis and, particularly, mitochondrial origins.

According to our results, there is a difference between trophic and catalytic reactions mediated by products in microbial relationships, yielding different dynamics and leading to different ecological and evolutionary coexistence. We have demonstrated that symmetric byproduct cross-feeding (mutual consumption of waste) is the most stable interaction of two species, and that asymmetric, unidirectional cross-feeding likely evolves toward symmetric, reciprocal interaction.

Our main conclusion is that nutritional cross-feeding can be stable both in the short and long terms, where selfish mutants cannot generally invade the mutual pair once it has established. Interactions are immediately beneficial for both parties, as the invested cost of disposing the waste is returned by the partner removing the inhibitor on growth. Cheaters stealing public goods do not affect this private benefit. Therefore, as we have shown, evolution leads to increased efficiency of using the byproduct of the partner. This kind of stable, mutual partnership rests on our assumptions of independent resources for two (and only two) species and stable flux for both resources. From a strictly dynamical point of view, the more symmetric sulphur cycling^78^ is more stable than the hydrogen^70^ or reverse flow hypotheses^69^, but this comparison ignores all other relevant aspects (like phylogenetic affiliations, absolute energetic costs and benefits).

Our second conclusion is that while a cross-facilitative interaction is stable ecologically, it can be easily destroyed by selfish mutants. If producing the enzyme is costly, and it does not provide any significant private benefit (but benefits everyone), then cross-facilitation is susceptible for exploitation and a selfish mutant can invade and destroy the interaction, as we have demonstrated, in line with what the “tragedy of the commons”^50^ imply. Consequently, it has been argued that a private benefit of the producer is necessary for cooperators to successfully withstand the invasion of cheaters^23,83^. In our model of cross-facilitation, there is no additional private benefit, while in cross-feeding, the private benefit is the disposal of toxic waste.

A trivial mechanism to maintain evolutionary stability of cooperation is spatial aggregation^84,85^. Microbes rarely interact exclusively in well mixed environments, they rather form dense, spatially structured, inhomogeneous communities in most habitat. In such aggregations (e.g. biofilms), interactions are localized and neighbourhoods are stable for a longer time^1,86^, which may increase the private benefit of catalytic products. Cooperative groups are less susceptible for cheaters and the longer timespan of interactions may facilitate evolutionary stability both for cross-feeding and collaborative-feeding for auxotrophic dependencies to develop. While we have demonstrated here the potential of cross-feeding for stable symbiosis and eukaryogenesis, collaborative-feeding (or more generally, cross-facilitation) may have also been relevant to such processes when such facilitating factors (like spatial inhomogeneities) are provided – but this claim must be examined more scrupulously via modelling.

Carefully constructed mathematical models may expose hidden assumptions or nontrivial dynamics, which verbal models may miss or obscure. Our models demonstrate that ecologically stable coexistence of a syntrophic pair does not ensure their evolutionary stability (just as metabolic compatibility does not necessarily entail increased growth or synergies^61^). In the cross-facilitation case in particular, an initially mutualistic interaction degrades over evolutionary time as selfish cheaters and free-riders invade the system. Most of the syntrophic hypotheses of mitochondrial origin assume an asymmetric setup at the origin. As our results demonstrate, nutritional cross-feeding, even unilateral, can lead to stable syntrophic mutualism. However, it remains an open question whether the initial mitochondriogenetic syntrophy was symmetric^71,78^ or asymmetric^69,70^.

On a final note, one cannot exclude that non-mutual syntrophy lead to endosymbiosis, i.e. symbiont uptake came *before* mutualism. However, one can convincingly argue against it, as we already did^10^: once inside the host, genetic, dynamical and cell-cycle synchronization issues readily arise that would provide ample cause for exploited parties to disrupt the partnership. We believe that if the initial interaction was metabolic syntrophy, the merger likely happened *after* cross-feeding become symmetric and mutual (unless there were other factors in play, e.g. phagocytosis^74^). If syntrophy was already mutually beneficial, then it is of both parties’ interest to sort out dynamical issues (via e.g., central control) when being physically integrated. We do not see examples to either scenario, but both should be tested in the lab.

## Methods

### Model description

We model the dynamics of interacting species via resource-consumer dynamics. There are two primary resources ${R}_{1}$ and ${R}_{2}$ with concentrations ${\rho }_{1}$ and ${\rho }_{2}$, and three consumer species ${N}_{1}$, ${N}_{2}$, and ${N}_{3}$ with densities ${n}_{1}$, ${n}_{2}$, and ${n}_{3}$. Species 1 consumes resource ${R}_{1}$ only, while species 2 and 3 compete for ${R}_{2}$; that is, they are neither depending on each other nor are in direct competition for the resource. In the eukaryogenetic context it is generally assumed that ancestral partners had substantially different metabolisms, relying on different resources, as they belonged to different domains. Additionally, species 1 produces ${X}_{1}$ with concentration ${x}_{1}$, and species 2 produces a different product ${X}_{2}$ with concentration ${x}_{2}$. Species 3 is a potential selfish mutant of species 2, feeding on ${R}_{2}$ (Fig. 3).

In the case of cross-feeding, ${X}_{i}$ is a byproduct waste metabolite that is consumed by the other species (Fig. 1A). For cross-facilitation, ${X}_{i}$ is an enzyme that is not consumed but remains reusable within the local environment^19^. We assume that the catalytic product is costly to produce, because it is a larger molecule that requires active secretion by the producer (e.g., catalytic enzymes that digest resources externally^4,21–23^). For the sake of simplicity, we assume ${X}_{i}$ is an external enzyme that improves resource consumption for all species (Fig. 1B). It is unlikely that such a costly enzyme is externalized for the sole benefit of others, hence we assume that there is always some private benefit associated with production (see the annamox community in Table S1 for a counterexample). Otherwise, it would always be easy for selfish mutants to benefit from the product without reciprocation, which would leave the producer to pay all the cost of production without any hope for benefit, leading to the disruption of cooperation^24,51^.

**Figure 7:**
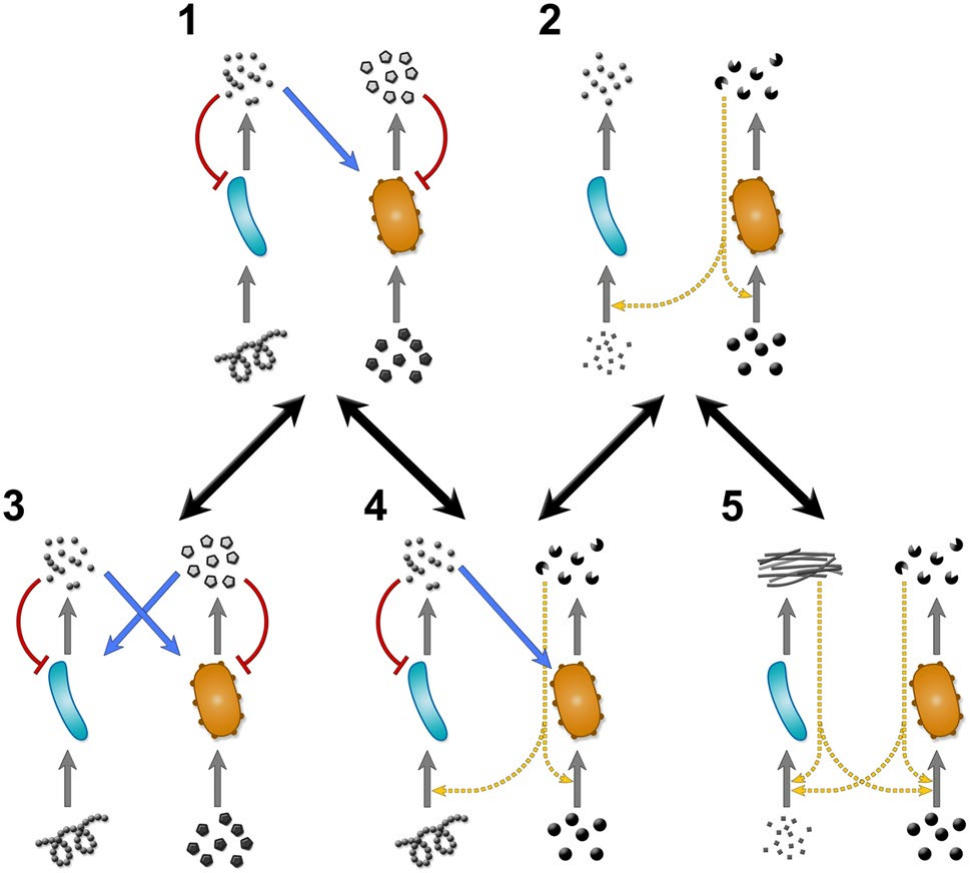
Possible evolutionary transitions (thick black arrows) between different syntrophic interaction topologies of cross-feeding (1, 3, 4) and collaborative feeding (cross-facilitation) (2, 5, 4). Grey and blue arrows indicate trophic interactions, red curves indicate inhibitory and dashed yellow arrows catalytic interactions. There are other possible cases not displayed, but we assume that further topologies are isomorphic to displayed ones up to a symmetric re-labelling of species and interactions. We omit cases where a species simultaneously exerts both a trophic and a catalytic effect on the partner, as that would require multiple products of the same species and thus lead to topologies that are not isomorphic (up to a re-labelling of species) to any of the depicted ones. Different mutants can appear, inheriting the interaction topology of either species 1 or 2. Panel 1 corresponds to flow-through syntrophy, while panel 3 may represents the recycle type if the end products $x_{1}$ and $x_{2}$ represent different states of the same molecule and are stoichiometrically coupled (cf. Fig. 6). Transformative interactions (normal arrows) are not expected to evolve to catalytic ones, or vice versa. We have explicitly tested transitions $1 \leftrightarrow 3$ and $2\leftrightarrow5$ in the main text (and $1\leftrightarrow4$ in Supplementary Information SI 14).

Figure 7 displays the potential evolutionary transitions that can happen in a syntrophic pair. We have explicitly investigated transitions 1–3 and 2–5 (SI 8 and 12). We note that all parameters used in the model are assumed to be nonnegative (for parameters, see Supplementary Table S2).

### Model of cross-feeding

We can safely assume fast resource dynamics compared to the dynamics of the species. From this, the resource densities ${\rho }_{1}$ and ${\rho }_{2}$ can be expressed directly as

1 $$\begin{aligned} {\rho }_{1}&={r}_{1}-{c}_{1}{n}_{1}, \\ {\rho }_{2}&={r}_{2}-{c}_{2}{n}_{2}-{c}_{3}{n}_{3}.\end{aligned}$$

Here ${r}_{i}$ is the maximum, unconsumed equilibrium resource density, and the ${c}_{i} {n}_{i}$ terms measure how much of resource is locked up in the biomass of species $i$. In turn, the abundances follow simple consumer-resource dynamics based on the model of^52^, auxiliated with the growth benefit received from the metabolic byproducts, with conversion efficiency ${g}_{i}$ for species $i$. Furthermore, byproduct ${X}_{i}$ is considered a waste product that can accumulate and inhibit the metabolism of species $i$ in proportion to the concentration of ${X}_{i}$. Therefore, the mortality rate of species $i$ is increased by ${h}_{i}{x}_{i}$, where all the ${h}_{i}$ are positive constants that describe the strength of inhibition. Thus, the dynamical equations for these three species are as follows:

2 $$\begin{aligned} \frac{\mathrm{d}{n}_{1}}{\mathrm{d}t} & ={n}_{1}\left({b}_{1}{\rho }_{1}+{g}_{1}{x}_{2}-{d}_{1}-{h}_{1}{x}_{1}\right), \\ \frac{\mathrm{d}{n}_{2}}{\mathrm{d}t}& ={n}_{2}\left({b}_{2}{\rho }_{2}+{g}_{2}{x}_{1}-{d}_{2}-{h}_{2}{x}_{2}\right), \\\frac{\mathrm{d}{n}_{3}}{\mathrm{d}t} &={n}_{3}\left({b}_{3}{\rho }_{3}+{g}_{3}{x}_{1}-{d}_{3}-{h}_{3}{x}_{2}\right), \end{aligned}$$

where ${b}_{i}$ is the conversion constant of resources into the reproduction of species $i$, and ${d}_{i}$ is the natural death rate of species $i$. Here we assume that apart from ${\rho }_{i}$, ${x}_{j}$ is used as an additional resource for species $i$. Note, that species 3 not only competes with species 2 for ${\rho }_{2}$, but may also benefit from the product ${x}_{1}$ produced by species 1. Also, for any species $i$, the combination ${b}_{i}=0$ and ${g}_{i}>0$ represents obligate dependence on the partner. We ignore this situation, assuming that species are initially free living, and do not depend on obligate metabolic partners.

Due to the fast resource dynamics, we can substitute the expressions for the resources [Eq. (1)] into the above system and rearrange:

3 $$\begin{aligned} \frac{\mathrm{d}{n}_{1}}{\mathrm{d}t}&={n}_{1}\left(\left({b}_{1}{r}_{1}-{d}_{1}\right)+{g}_{1}{x}_{2}-{b}_{1}{c}_{1}{n}_{1}-{h}_{1}{x}_{1}\right), \\ \frac{\mathrm{d}{n}_{2}}{\mathrm{d}t}&={n}_{2}\left(\left({b}_{2}{r}_{2}-{d}_{2}\right)+{g}_{2}{x}_{1}-{b}_{2}{c}_{2}{n}_{2}-{b}_{2}{c}_{3}{n}_{3}-{h}_{2}{x}_{2}\right), \\ \frac{\mathrm{d}{n}_{3}}{\mathrm{d}t}&={n}_{3}\left(\left({b}_{3}{r}_{2} -{d}_{3}\right)+{g}_{3}{x}_{1}-{b}_{3}{c}_{2}{n}_{2}-{b}_{3}{c}_{3}{n}_{3}-{h}_{3}{x}_{2}\right).\end{aligned}$$

In turn, the metabolite dynamics read

$$\begin{aligned} \frac{\mathrm{d}{x}_{1}}{\mathrm{d}t}&={k}_{1}{n}_{1}-\left({w}_{2}{n}_{2}{x}_{1}+{w}_{3}{n}_{3}{x}_{1}\right)-{\delta }_{1}{x}_{1}, \\ \frac{\mathrm{d}{x}_{2}}{\mathrm{d}t}&={k}_{2}{n}_{2}-{w}_{1}{n}_{1}{x}_{2}-{\delta }_{2}{x}_{2},\end{aligned}$$

where ${k}_{i}$ is the production rate and ${w}_{i}$ is the consumption rate of byproduct of species $i$, and ${\delta }_{i}$ is the rate of decomposition. If we additionally assume fast dynamics for the ${x}_{i}$ as well, we can set $\frac{\mathrm{d}{x}_{i}}{\mathrm{d}t}=0$ to get the following quasi-equilibrium equations:

$$\begin{aligned} {x}_{1}&=\frac{{k}_{1}{n}_{1}}{ {w}_{2}{n}_{2}+{w}_{3}{n}_{3}+{\delta }_{1}}, \\ {x}_{2}&=\frac{{k}_{2}{n}_{2}}{{w}_{1}{n}_{1}+{\delta }_{2}}.\end{aligned}$$

Substituting these back into [Eq. (3)] yields:

4 $$\begin{aligned}\frac{\mathrm{d}{n}_{1}}{\mathrm{d}t}&={n}_{1}\left(\left({b}_{1}{r}_{1}-{d}_{1}\right)+{g}_{1}\frac{{k}_{2}{n}_{2}}{{w}_{1}{n}_{1}+{\delta }_{2}}-{b}_{1}{c}_{1}{n}_{1}-{h}_{1}\frac{{k}_{1}{n}_{1}}{{w}_{2}{n}_{2}+{w}_{3}{n}_{3}+{\delta }_{1}}\right), \\ \frac{\mathrm{d}{n}_{2}}{\mathrm{d}t}&={n}_{2}\left(\left({b}_{2}{r}_{2}-{d}_{2}\right)+{g}_{2}\frac{{k}_{1}{n}_{1}}{{w}_{2}{n}_{2}+{w}_{3}{n}_{3}+{\delta }_{1}}-{b}_{2}{c}_{2}{n}_{2}-{b}_{2}{c}_{3}{n}_{3}-{h}_{2}\frac{{k}_{2}{n}_{2}}{{w}_{1}{n}_{1}+{\delta }_{2}}\right), \\ \frac{\mathrm{d}{n}_{3}}{\mathrm{d}t}&={n}_{3}\left(\left({b}_{3}{r}_{2}-{d}_{3}\right)+{g}_{3}\frac{{k}_{1}{n}_{1}}{{w}_{2}{n}_{2}+{w}_{3}{n}_{3}+{\delta }_{1}}-{b}_{3}{c}_{2}{n}_{2}-{b}_{3}{c}_{3}{n}_{3}-{h}_{3}\frac{{k}_{2}{n}_{2}}{{w}_{1}{n}_{1}+{\delta }_{2}}\right). \end{aligned}$$

### Model of cross-facilitation

In the catalytic case, metabolite ${X}_{i}$ represents a catalytic factor (such as an enzyme) that is costly to externalize but has a positive effect on resource consumption both for the producer and its competitor. Consequently, all products help all species, and they do not impose (self-)inhibition. For a different catalytic topology where enzyme ${X}_{i}$ enables resource ${R}_{i}$ only but makes it available for all species, see SI 13.

Motivated by Michaelis–Menten enzyme kinetics, we use the standard saturation functions for enzymes. We rely on the fast dynamics of resources, just as before, using [Eq. (1)]. Accordingly, the equations for cross-facilitation on ${X}_{1},{X}_{2}$ are:

5 $$\begin{aligned}\frac{\mathrm{d}{n}_{1}}{\mathrm{d}t}&={n}_{1}\left({\rho }_{1}\left({b}_{1}+\frac{{s}_{11}{x}_{1}}{{m}_{1}+{x}_{1}}+\frac{{s}_{12}{x}_{2}}{{m}_{1}+{x}_{2}}\right)-{d}_{1}\right), \\ \frac{\mathrm{d}{n}_{2}}{\mathrm{d}t}&={n}_{2}\left({\rho }_{2}\left({b}_{2}+\frac{{s}_{21}{x}_{1}}{{m}_{2}+{x}_{1}}+\frac{{s}_{22}{x}_{2}}{{m}_{2}+{x}_{2}}\right)-{d}_{2}\right), \\ \frac{\mathrm{d}{n}_{3}}{\mathrm{d}t}&={n}_{3}\left({\rho }_{2}\left({b}_{3}+\frac{{s}_{31}{x}_{1}}{{m}_{3}+{x}_{1}}+\frac{{s}_{32}{x}_{2}}{{m}_{2}+{x}_{2}}\right)-{d}_{3}\right),\end{aligned}$$

where ${m}_{i}$ is the Michaelis constant where the reaction rate is at its half-maximum, and ${s}_{ij}$ defines the maximum rate of conversion of enzyme $j$ by species $i$. Since enzymes are not consumed, their concentrations are determined by the balance of production and spontaneous decay. The first is proportional to the concentration of producer strains, the latter to the actual concentration of the enzyme:

$$\begin{aligned}\frac{\mathrm{d}{x}_{1}}{\mathrm{d}t}&={k}_{1}{n}_{1}-{\delta }_{1}{x}_{1}, \\ \frac{\mathrm{d}{x}_{2}}{\mathrm{d}t}&={k}_{2}{n}_{2}+{k}_{3}{n}_{3}-{\delta }_{2}{x}_{2}.\end{aligned}$$

We again assume fast dynamics for ${X}_{i}$, leading to ${x}_{1}=\frac{{k}_{1}}{{\delta }_{1}}{n}_{1}, {x}_{2}=\frac{{k}_{2}{n}_{2}+{k}_{3}{n}_{3}}{{\delta }_{2}}$ as above. Substituting these into [Eq. (5)], the model is as follows:

6 $$\begin{aligned}\frac{\mathrm{d}{n}_{1}}{\mathrm{d}t}&={n}_{1}\left({(r}_{1}-{c}_{1}{n}_{1})\left({b}_{1}+\frac{{s}_{11}{k}_{1}{n}_{1}}{{m}_{1}{\delta }_{1}+{k}_{1}{n}_{1}}+\frac{{s}_{12} \left({k}_{2}{n}_{2}+ {k}_{3}{n}_{3}\right)}{{m}_{1}{\delta }_{2}+{{k}_{2}n}_{2}+{k}_{3}{n}_{3}}\right)-{d}_{1}\right), \\ \frac{\mathrm{d}{n}_{2}}{\mathrm{d}t}&={n}_{2}\left({(r}_{2}-{c}_{2}{n}_{2}-{c}_{3}{n}_{3})\left({b}_{2}+\frac{{s}_{21}{k}_{1}{n}_{1}}{{m}_{2}{\delta }_{1}+{k}_{1}{n}_{1}}+\frac{{s}_{22} \left({{k}_{2}n}_{2}+{{k}_{3}n}_{3}\right)}{{m}_{2}{\delta }_{2}+{k}_{2}{n}_{2}+{k}_{3}{n}_{3}}\right)-{d}_{2}\right),\frac{\mathrm{d}{n}_{3}}{\mathrm{d}t}={n}_{3}\left({(r}_{2}-{c}_{2}{n}_{2}-{c}_{3}{n}_{3})\left({b}_{3}+\frac{{s}_{31}{k}_{1}{n}_{1}}{{m}_{2}{\delta }_{1}+{k}_{1}{n}_{1}}+\frac{{s}_{32} \left({{k}_{2}n}_{2}+{{k}_{3}n}_{3}\right)}{{m}_{2}{\delta }_{2}+{{k}_{2}n}_{2}+{k}_{3}{n}_{3}}\right)-{d}_{3}\right),\end{aligned}$$

### Adaptive dynamics

To simulate the evolution of cross-feeding and cross-facilitation between the different species, we implemented a numerical version of adaptive dynamics^57^. First, we assume that there is an underlying trait, $z$, whose value determines the death rates $d$, and the efficiency of byproduct consumption $g$ (or in case of cross-facilitation, the production efficiency $k$). Second, we assume that there is a trade-off between these quantities: a higher byproduct consumption (or enzyme production) efficiency can only be attained at the cost of increased mortality rates. Such a trade-off can be implemented via the following equations (for details, see SI 7):

7 $$\begin{aligned}g\left(z\right)&=\frac{1}{2}\left(1+\mathrm{tanh}\left(\frac{z-\overline{z}}{\sigma }\right)\right), \\ d\left(z\right)&=\eta \mathrm{max}\left(0,z\right).\end{aligned}$$

With these choices, the uptake rates $g(z)$ vary between 0 and 1, approaching 0 for very large negative $z$ and approaching 1 for very large positive $z$, following a sigmoidal curve (Fig. S5). This function therefore expresses the fact that uptake rates cannot be increased ad infinitum, so that there are diminishing returns on increasing $z$ beyond some point. In turn, the death rates $d(z)$ increase linearly with $z$, but only as long as they are positive. When they hit zero (in our parameterization, this happens exactly when $z=0$), the death rates stay at their lowest biologically meaningful value of 0 and no longer change.

Simulated evolution then proceeds by first initializing species 1 and 2 with different $z$ values, but with species 2 starting out with a low *z* (implying negligibly low cross-feeding). In the basic version of the simulation, only species 2 evolves. We checked what happens when both species evolve and get qualitatively the same results. Now we generate a random mutant whose trait is similar to that of the original species 2 and introduce it into the community at a low initial density. We then run the dynamics until we reach equilibrium, at which point we remove those species whose densities dropped below an extinction threshold. Of the remaining mutants, we pick one, randomly mutate its trait value, and introduce a new mutant with that trait and a small invasion density—and so on. After several iterations of this procedure, we end up with a community potentially consisting of several species. For further details, see SI 7, 8 and 12. The code to reproduce the adaptive dynamics results is included as supplementary material (see SI 15).

## Supplementary Information

Supplementary Information 1.

Supplementary Information 2.

## Data Availability

All data generated or analysed during this study can be reproduced with the code provided as a set of Supplementary Information files (see SI 15).

## Code availability

The code to reproduce all the data (and figures) generated and analysed during this study (up to a change in the seed of the pseudorandom number generators), is provided as a set of Supplementary Information files (see SI 15).
