# Supplementary material for: Eco-evolutionary modelling of microbial syntrophy indicates the robustness of cross-feeding over cross-facilitation
Source: Sci Rep. 2023 Jan 17;13:907. doi: 10.1038/s41598-023-27421-w (PMC9845244; doi:10.1038/s41598-023-27421-w)
Supplement: Supplementary file 1 — Supplementary Information 1. [file 41598_2023_27421_MOESM1_ESM.pdf]

Supplementary information: Eco-evolutionary modelling of microbial syntrophy indicates the robustness of cross-feeding over cross-facilitation

Boza, G.[1,2,3]*, Barabás, G.[4,1]*, Scheuring, I.[1] & Zachar, I.[1,5,6§]

[1] Institute of Evolution, MTA Centre for Ecological Research, Budapest, Hungary
[2] ASA Program, International Institute for Applied Systems Analysis (IIASA), Laxenburg, Austria
[3] Centre for Social Sciences, Budapest, Hungary
[4] Division of Ecological and Environmental Modeling, Linköping University, Linköping, Sweden
[5] Department of Plant Systematics, Ecology and Theoretical Biology, Eötvös Loránd University, Budapest, Hungary
[6] Parmenides Foundation, Centre for the Conceptual Foundation of Science, Pullach im Isartal, Germany

* Shared first authorship
§ Corresponding author

Contents

1. Examples of syntrophy ... 2
2. Model parameters ... 4
3. Existence of a single, globally stable internal fixed point for cross-feeding without inhibition ... 5
4. Numerical investigation of stable fixed points for cross-feeding with inhibition ... 7
5. Existence of a single, globally stable internal fixed point for cross-feeding with inhibition ... 7
6. Condition of invasion for a third species for cross-feeding ... 8
7. Adaptive dynamics for cross-feeding without inhibition ... 10
8. Adaptive dynamics for cross-feeding with inhibition ... 14
9. Coexistence of species 1 and 2 for cross stimulation ... 14
10. Numerical investigation of stable fixed points for cross-stimulation ... 15
11. Existence of a single, globally stable internal fixed point for cross-stimulation ... 15
12. Adaptive dynamics for cross-stimulation ... 16
13. Adaptive dynamics for another model of cross-stimulation ... 17
14. Adaptive dynamics for a hybrid cross-feeding, cross-stimulation system ... 19
15. Code to reproduce analyses and figures ... 21
16. Supplementary references ... 22

1. Examples of syntrophy

Table S1. Examples of prokaryotic and eukaryotic syntrophies. Column N indicates the number of species involved in the interaction (species count within the community). Column N/E indicates if the interaction is naturally appearing (N) or engineered (E). Interaction type can be cross-feeding (CF) or cross-facilitation (CX) or hybrid, that is, both cross-feeding and cross-facilitation are present.

Description of the interaction	*N*	N/E	Type	Ref.
Cross-feeding between auxotrophic *E. coli* knock-out mutants lacking genes required for the biosynthesis of amino acids, nucleotides, and cofactors, as well as genes involved in glycolysis and respiration. In an experiment, 17% of random pairings out of 1035 combinations showed synergetic growth in co-culture of such mutants.	2	E	CF flow-through	1
Multi-member cross-feeding between engineered amino-acid auxotroph *E. coli* mutants. Biosynthetically costly amino acids tend to promote stronger cooperative interactions than cheaper ones. The evolved cross-feeding pair was also resistant against the invading autotroph wild-type strain. Besides pairwise, three-member communities (with double-auxotrophs) also showed synergistic growth when all three members were present. In another experiment, an initially 14-member systems undergo a drastic population shift toward a consortium dominated by four members, hence showing that microbes with multi-auxotrophic phenotypes can stably evolve, but only up to a limited size of the interaction network.	2…14	E	CF flow-through	2–4
Laboratory-evolved cooperation between *Salmonella enterica* and an auxotrophic *E. coli* strain. *Salmonella* consumes metabolic waste from *E. coli*, while *E. coli* relies on *Salmonella* to synthesize the essential amino acid methionine.	2	E	CF flow-through	5
Anaerobic methanotrophic archaea in a syntrophic relationship with sulfate-reducing bacteria. The partners rely on an efficient electron exchange via reducing equivalents or direct, tubular cell–to-cell connections.	2	N	CF recycle	6,7
Successional colonization dynamics is often driven by cross-feeding where pioneer primary degraders enable late colonizers to feed on their by-products. For example, in particle-attached biofilms that degrade insoluble forms of organic matter concentrated on particles, community assembly is driven by a functional characteristic-based trophic structure: from narrow-range degraders to broad-range consumers, via resultant by-products of the degradation process.	2+	N	CF flow-through	8–10
The majority of bacterial species produce siderophores, a central component in the mechanism of iron scavenging. Siderophores are costly products secreted to make iron acquisition possible, yet the iron made available can be taken up by non-producers as well. After the iron is imported to the cell, the extracellular siderophore can be recycled (salvaged) or undergo hydrolysis.	2+	N	CX	11
Collective resistance can emerge in a community if an individual strain provides resistance for all members. It does so either by secreting extracellular enzymes to neutralize antibiotics (e.g. β-lactamase) or by neutralizing them intracellularly hence removing them from the environment. A particular form is the so-called cross-protection mutualism, in which sensitive and resistant strains in coculture can protect each other in a multidrug environment.	2+	N	CX	12–15
The yeast *Saccharomyces cerevisiae* secretes invertase enzyme to hydrolyze sucrose. Almost 99% of the end products, the monosaccharides, diffuse away benefiting other species nearby.	2+	N	CX	16
The biofilm can provide protection against antibiotics originating from outside of the community, due to complex tolerance and resistance mechanisms, that may be more effective than resistance due to extracellular enzymes. The physical separation of the cells within the biofilm matrix from the external environment provides efficient protection against predation, invasion, bacteriophages, antibiotics, etc.	2+	N	CX	17–19
Surfactin bolsters surface spreading by reducing surface tension and promoting filamentous growth, benefiting all cells in the vicinity. It is costly to produce, hence acts like a common good, exploitable by cheaters.	2+	N, E	CX	20
Pathogenicity in microbes is most often carried out by the secretion and acquisition of extracellular molecules that help to subvert the immune response of the host or facilitate the access to host nutrients, for example by triggering the outflow from the host. These products enable surrounding microbes to get access to energy rich nutrients of the host, acting as public goods that facilitate higher nutrient uptake for beneficiaries.	2+	N	CX	21,22
Most bacterial species depend on cofactors acquired from the environment via cross-feeding, which they are incapable of producing due to their costly and complicated synthesis. It is predicted that the majority of bacteria harbor enzymes dependent on the cofactor corrinoid, which they have to acquire via cross-feeding, and which that enable pathways that significantly enhance their metabolic capacity.	2+	N	CX	23

Rhodococcus species can degrade recalcitrant toxic pollutants in diverse environments, but their degrading efficiency is severely reduced by various environmental stress. *Bacillus cereus* exhibits strong stress resistance and can regulate pH. For example, *Rhodococus ruber* degrades a tetrahydrofuran providing acidic metabolites to *B. cereus*, which, in return, regulates pH and secretes nutrients essential for *R. ruber.*	2	N	hybrid, flow-through	24
Up to five-membered cellulose-degrading community of various strains coexist stably under laboratory conditions exhibiting a complex, intertwined interaction network with mutualistic or commensalistic (syntrophy), inhibitory (as the accumulating intermediary product inhibits the catalytic activity of species), and competitive relationships. Both cross-feeding and cross-facilitative interactions are at play.	5	E	hybrid, flow-through	25
Certain bacteria (e.g. Bacteroidales) live in the human intestine capable of breaking down polysaccharides extracellularly using glycoside hydrolases that in some cases are secreted outside of the cell. This breaking down is costly and results in diffusible public goods that benefit other species. For example, *Bacteroides ovatus* digests polysaccharides extracellularly, apparently benefiting only other species, and hence can be seen as an altruist act. The benefit of this act is enjoyed by nearby species, such as *Bacteroides vulgatus*, which reciprocates in various ways, including detoxification of inhibitory substances, or production of a growth promoting factor, initiating defense mechanisms and metabolism. This implies that this naturally evolved system involves both cross-feeding (by *B. ovatus*) and cross-facilitative interactions (by *B. vulgatus*).	2+	N	hybrid, flow-through	26–35
The bacteria *Dehalococcoides mccartyi* is the only known species that can completely degrade trichloroethene (TCE) from contaminated groundwater, but it depends on other species for a variety of exogenous compounds, such as hydrogen, acetate, corrinoids, biotin, and thiamine. Several other compounds, including hydrogen and CO, is hypothesized to take part in the syntrophic interactions.	2+	N	hybrid, flow-through	36
The consortium *Chlorochromatium aggregatum* consists of a motile central betaproteobacterium which transports its non-motile photosynthetic epibionts (*Chlorobium*) towards light while the epibionts provide energy for the host. Several other consortia are described with similar structures. These examples are the closest to syntrophic endosymbiosis and demonstrate the most sophisticated forms of pairwise prokaryotic ectosymbioses.	2	N	hybrid, flow-through	37–39
In a metal working fluid-degrading community of low diversity (consisting of *Agrobacterium tumefaciens*, *Comamonas testosteroni*, *Microbacterium saperdae*, and *Ochrobactrum anthropi*), facilitative or neutral interactions dominate in a toxic environment. *M. saperdae* is highly dependent on by-products of other community members (cross-feeding), whereas cross-facilitative interactions arose by the other species removing toxic compounds initially present in the environment, enabling growth for the others.	4	N	hybrid	40
Anammox communities mediate anaerobic ammonium oxidation (anammox) via complex interaction networks. Anammox granules consist of a mixture of cell aggregates and abiotic particles embedded within a matrix of organic extracellular polymeric substances (EPS). Within this medium, metabolite exchange takes place between heterotrophic and anammox bacteria. These include the products of anaerobic ammonium oxidation, nitrite loop as well as vitamins, and extracellularly degraded proteins. In particular, a *Brocadia* species demonstrating increased expression of genes involved in pathways for B1, B7 and B12 synthesis may indicate that this strain provides the above vitamins for the whole community.	5+	N	hybrid, recycle	41–43

2. Model parameters

Table S2. Parameters of the models with some default values. Note that cross-feeding and cross-facilitation models use slightly different sets of parameters. All parameters are non-negative, by definition.

**cross-feeding**		**cross**-facilitation
**resource (R) properties**		
density of resource R_i	ρ_i	density of resource R_i
maximum, unconsumed equilibrium density of resource R_i	r_i	maximum, unconsumed equilibrium density of resource R_i
ratio of resource R_i locked up in the biomass	c_i	ratio of resource R_i locked up in the biomass
**species (N) properties**		
density of species N_i	n_i	density of species N_i
conversion constant of resource R_i into reproduction of species N_i	b_i	conversion constant of resource R_i into reproduction of species N_i
spontaneous death rate of species N_i	d_i	spontaneous death rate of species N_i
conversion efficiency of byproduct X_j by species N_i	g_i	–
strength of inhibition on species N_i by the byproduct X_i	h_i	–
consumption rate of any byproduct by species N_i (uniform for all byproducts)	w_i	–
–	m_i	amount of enzyme where the reaction rate is at half-maximum of species N_i (Michaelis constant)
–	s_{ij}	maximum rate of conversion of metabolite X_j to reproduction of species N_i
evolutionary trait of species N_i, defining d_i and k_i, being in trade-off	z_i	evolutionary trait of species N_i, defining d_i and k_i, being in trade-off
production rate of metabolite X_i by species N_i	k_i	production rate of enzyme X_i by species N_i
**product (X) properties**		
density of metaboliteX_i	x_i	density of enzyme X_i
decomposition rate of metabolite X_i	δ_i	decomposition rate of enzyme X_i
**adaptive dynamics**		
mutant invasion density	$n_{inv} = 1$	mutant invasion density
mutant invasion time (after last equilibrium)	$t_{inv} = 10^4$	mutant invasion time (after last equilibrium)
SD of normal distribution centred at trait when mutating	$\mu_{SD} = 10^{-2}$	SD of normal distribution centred at trait when mutating
extinction density threshold	$n_\theta = 10^{-3}$	extinction density threshold
iteration count	$G = 300$	iteration count
**trade-off**		
expected mean of trait z	$\bar{z} = 0.5$	expected mean of trait z
steepness of the sigmoid transition curve	$\sigma = 1/5$	steepness of the sigmoid transition curve
scaling factor	$\eta = 0.1$	scaling factor

3. Existence of a single, globally stable internal fixed point for cross-feeding without inhibition

We assume that the inhibitory effect of externalized waste product X_i over the producer species i (or anyone) can be neglected in the system described by (Eq. 4) in the main text. That is $h_i = 0$ for all species i (see Figure S1). The internal fixed points can be calculated analytically for two species (1 and 2), but their complexity prevents effective analysis of the system. Therefore, we rely on other means to ascertain that there is a single internal fixed point which always exists, and that it is stable.

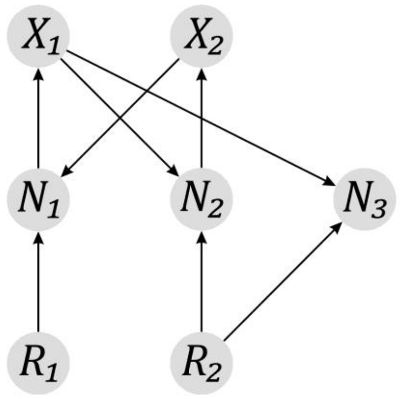

Figure S1. Model of cross-feeding without self-inhibition, with mutant species N_3. Arrows indicate consumption and production.

Without species 3, the system simplifies to:

$$\frac{\mathrm{d}n_1}{\mathrm{d}t} = n_1\left((b_1 r_1 - d_1) + g_1 \frac{k_2 n_2}{w_1 n_1 + \delta_2} - b_1 c_1 n_1\right),$$
$$\frac{\mathrm{d}n_2}{\mathrm{d}t} = n_2\left((b_2 r_2 - d_2) + g_2 \frac{k_1 n_1}{w_2 n_2 + \delta_1} - b_2 c_2 n_2\right).$$

(Eq. S1)

The per capita growth rates G_i are:

$$G_1 = (b_1 r_1 - d_1) + g_1 \frac{k_2 n_2}{w_1 n_1 + \delta_2} - b_1 c_1 n_1$$
$$G_2 = (b_2 r_2 - d_2) + g_2 \frac{k_1 n_1}{w_2 n_2 + \delta_1} - b_2 c_2 n_2.$$

(Eq. S2)

When $\frac{\mathrm{d}n_i}{\mathrm{d}t} = 0$, we get the zero net growth isoclines (ZNGI) from G_1, G_2 respectively, depending on n_1:

$$n_2 = \frac{(d_1 - b_1 r_1 + b_1 c_1 n_1)(\delta_2 + w_1 n_1)}{g_1 k_2},$$
$$n_2 = \frac{-d_2 w_2 + b_2 r_2 w_2 - b_2 c_2 \delta_1 + \sqrt{(-d_2 w_2 + b_2 r_2 w_2 + b_2 c_2 \delta_1)^2 + 4 b_2 c_2 g_2 k_1 w_2 n_1}}{2 b_2 c_2 w_2},$$

where we have ignored the irrelevant negative branch for the second isocline. The first ZNGI has the following structure: $n_2 = -A^2 + B\,n_1 + C^2 n_1^2$, where A, B, and C are (combinations of) constants. Squaring them indicates when one is guaranteed to be positive. In turn, the second ZNGI has the form $n_2 = -D + \sqrt{E^2 n_1 + F^2}$, which is qualitatively the same as the first one, just mirrored along the $n_1 = n_2$ identity line. That is, $G_1 = 0$ and $G_2 = 0$ defines convex and concave strictly monotonous curves in

the n_1, n_2 plane. Thus, they intersect each other at most in two points, of which at most one can be stable. The isoclines are depicted in Figure S2A.

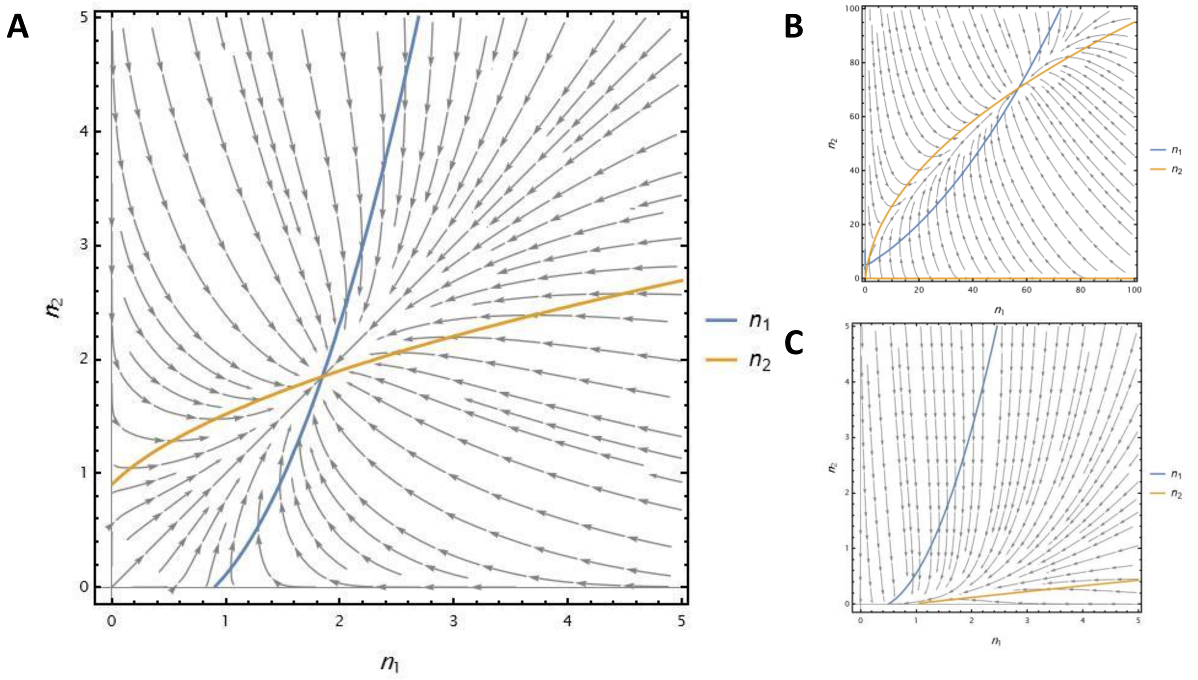

Figure S2. Zero net growth isoclines (blue for species N_1, yellow for N_2) over the phase plot of species 1 and 2 in the model of cross-feeding without inhibition. A: A single internal fixed point exists whenever species have positive growth rates when the partner is missing. The crossing of isoclines around {2, 2} indicates the stable internal fixed point, where species 1 and 2 can stably coexist. Parameters are $\{b_1 = b_2 = 1, r_1 = r_2 = 1, d_1 = d_2 = 0.1, c_1 = c_2 = 1, g_1 =, g_2 = 1, h_1 = h_2 = 0, \delta_1 = \delta_2 = 0.1, k_1 = k_2 = 1, w_1 = w_2 = 1\}$. B: If species cannot grow without each other, that is $r_i b_i < d_i$, two internal fixed points may exist of which one is stable only. However, such a case would imply that partners are obligately dependent on each other as they cannot grow without the partner. Parameters are the same as for panel A, with the following differences: $\{b_1 = b_2 = 0.01, \delta_2 = 5, g_1 = g_2 = 0.1, w_1 = w_2 = 0.1\}$. C: When the zero net growth isocline of n_1 crosses the $n_2 = 0$ line earlier than the zero net growth isocline of n_2, no internal fixed point exists (no crossing of the isoclines in the positive quadrant). Parameters are the same as for panel A, with the following differences: $\{d_1 = 0.5, d_2 = 10\}$.

We now see that there is at most one internal fixed point. If this fixed point exists, then it is locally stable, due to the geometric arrangement of the ZNGIs (n_1 increases to the left of the blue curve and decreases to the right of it; conversely, n_2 increases below the yellow curve and decreases above).

Assuming unrealistic parameter combinations, one can have two internal fixed points, of which only one would be stable (see Figure S2B). However, these cases imply that the per capita death rates are higher than the birth rates $r_i b_i < d_i$ (meaning that species are obligately dependent on each other), and/or an unrealistically high degradation rate of one of the byproducts.

Global stability can also be proved, using the Bendixson-Dulac criterion. This criterion states that, given some domain $\mathcal{D}$ in the phase plane, the system $n_1' = f(n_1, n_2), n_2' = g(n_1, n_2)$, and an arbitrary function $h(n_1, n_2)$, if the divergence $\partial_{n_1}(hf) + \partial_{n_2}(hg)$ is of the same sign everywhere in $\mathcal{D}$, then there can be no periodic orbits in that region. In this case, we can choose $h(n_1, n_2) = 1/(n_1\, n_2)$ to immediately prove the impossibility of cycles for the whole positive quadrant:

$$\nabla_{n_1,n_2} . \{hf, hg\} = \partial_{n_1}(hf) + \partial_{n_2}(hg) = -\frac{b_2 c_2}{n_1} - \frac{g_1 k_2 w_1}{(\delta_2 + w_1 n_1)^2} - \frac{b_1 c_1}{n_2} - \frac{g_2 k_1 w_2}{(\delta_1 + w_2 n_2)^2}.$$

Since all parameters are positive, this expression is always negative in the whole positive quadrant. Thus, equating $\mathcal{D}$ with this region, periodic solutions are impossible wherever $n_1 > 0$ and $n_2 > 0$.

Adding to this the fact that a two-dimensional differential equation system can never produce chaos, the only option left is that the locally stable fixed point must also be globally stable.

When does the fixed point exist? Nonexistence happens when the first isocline crosses the $n_2 = 0$ line earlier than the second isocline (for an example, see Figure S2C). In the biological context, this means that species 2 has too great of a mortality to persist, and only species 1 prevails.

4. Numerical investigation of stable fixed points for cross-feeding with inhibition

We examine the fixed point distribution for a large number of random parameter combinations for the equation system (Eq. 4) in the main text. For each combination, we perturb the parameter values $\{r_1 = r_2 = 1, \delta_1 = \delta_2 = 0.1, c_1 = c_2 = 1, b_1 = b_2 = 1, d_1 = d_2 = 0.01, k_1 = k_2 = 0.1, g_1 = g_2 = 0.1, h_1 = h_2 = 0.5, w_1 = w_2 = 1\}$ independently by adding a random value drawn from the normal distribution with mean 0 and standard deviation 0.5, ensuring that actual parameter values do not go below 10^{-3}. We calculate the numerical Jacobian of the system with the given parameters, substituting in the numerically calculated fixed points as the densities. For each fixed point, we check whether the eigen values of the Jacobian are all negatives. After extensive numerical testing (more than 100 000 independent random parameter combinations), we have not found a parameter combination which has more than 1 internal stable fixed point.

5. Existence of a single, globally stable internal fixed point for cross-feeding with inhibition

We now include self-inhibition of waste materials X_i on species N_i into the dynamics (see Figure S3).

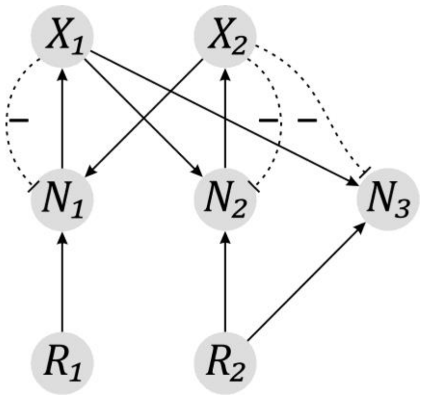

Figure S3. Model of cross-feeding with self-inhibition with mutant species N_3. Dashed arrows indicate self-inhibitory effect of produced metabolites. The mutant N_3 inherits the property of being inhibited by X_2 while it does not secrete it.

We follow the same analysis as before, in SI 3. The per-capita growth rates of the subsystem of species 1 and 2 are:

$$G_1 = b_1 r_1 - b_1 c_1 n_1 + g_1 \frac{k_2 n_2}{\delta_2 + w_1 n_1} - h_1 \frac{k_1 n_1}{\delta_1 + w_2 n_2} - d_1,$$

$$G_2 = b_2 r_2 - b_2 c_2 n_2 + g_2 \frac{k_1 n_1}{\delta_1 + w_2 n_2} - h_2 \frac{k_2 n_2}{\delta_2 + w_1 n_1} - d_2.$$

At the internal fixed point, where $n_1, n_2 > 0$:

$$G_1 = G_2 = 0.$$

Due to the complexities of the dynamics with self-inhibition, we cannot analytically or geometrically prove the existence of a single globally stable internal fixed point. However, based on the extensive

numerical investigations we have performed (see above), it is strongly believed that there is at most one internal stable fixed point of the 2-species system.

The same divergence formula can be used as before, to prove the impossibility of cycles for the whole positive quadrant ($n_1, n_2 > 0$), according to the Bendixson-Dulac criterion, assuming $n_1' = f(n_1, n_2), n_2' = g(n_1, n_2)$:

$$\partial_{n_1}(hf) + \partial_{n_2}(hg) = -\left(\frac{g_1 k_2 w_1}{(\delta_2 + w_1 n_1)^2}\right) - \frac{b_2 c_2 + \frac{h_2 k_2}{\delta_2 + w_1 n_1}}{n_1} - \frac{g_2 k_1 w_2}{(\delta_1 + w_2 n_2)^2} - \frac{b_1 c_1 + \frac{h_1 k_1}{\delta_1 + w_2 n_2}}{n_2}.$$

Since all parameters are positive, this expression is always negative in the whole positive quadrant. Thus, periodic solutions are impossible wherever $n_1, n_2 > 0$. Therefore, the locally stable fixed point must also be globally stable.

The zero net growth isoclines behave similarly to the system without self-inhibition, depicted in Figure S4. Again, biologically unrealistic parameters may result in the disappearance of the internal fixed point or the appearance of a second, instable internal fixed point, like without inhibition.

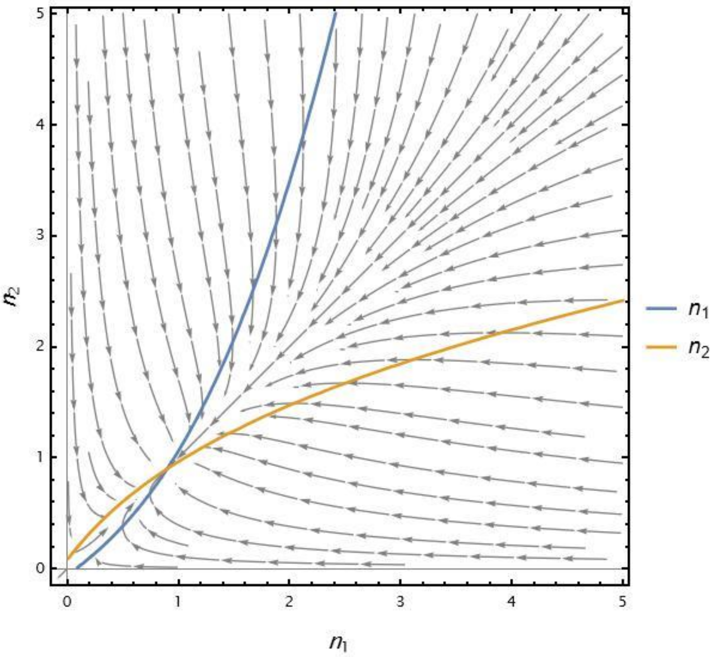

Figure S4. Zero net growth isoclines (blue and yellow) over the phase plot of species 1 and 2 in the model of cross-feeding with inhibition by the X_i. A: If species can grow without each other, that is $r_i b_i > d_i$, a single internal fixed point exists. The crossing of isoclines around $\{2, 2\}$ is the stable internal fixed point, where species 1 and 2 can stably coexist. Isoclines at the boundaries $n_1 = 0$ and $n_2 = 0$ are not shown but indicate trivial solutions where one or both species are extinct. Parameters are $\{b_1 = b_2 = 1, r_1 = r_2 = 1, d_1 = d_2 = 0.1, c_1 = c_2 = 1, g_1 = g_2 = 1, \delta_1 = \delta_2 = 0.1, k_1 = k_2 = 1, w_1 = w_2 = 1, h_1 = h_2 = 1\}$.

6. Condition of invasion for a third species for cross-feeding

We would like to know whether a mutant species can invade a presumably ecologically stable pair of cross-feeding species in case there is no inhibition by the products X_i. Therefore, we analyse the invasion dynamics of the model. We assume that species 1 and species 2 are present initially (Eq. S1), and we ignore self-inhibition of metabolites ($h_i = 0, \forall i$).

Above we have proven that there is only one stable positive fixed point of this system. Let us denote the fixed points of species 1 and 2 as $\hat{n}_1$ and $\hat{n}_2$, respectively. Because of the definition of fixed points, it is true that:

$$r_2 b_2 - d_2 + g_2 \frac{k_1 \hat{n}_1}{\delta_1 + w_2 \hat{n}_2} - b_2 c_2 \hat{n}_2 = 0.$$

(Eq. S3)

Let us use the following notations: $b_3 = b_2 + \Delta b,\ d_3 = d_2 + \Delta d,\ g_3 = g_2 + \Delta g,$ where the differences between the parameters b, d and g ($\Delta b, \Delta d$ and Δg respectively), can be negative positive or zero. Substituting (Eq. S3) into the dynamical equation of n_3 and taking the linear approximation of it by assuming that $n_3 \ll 1$:

$$\frac{dn_3}{dt} = \left(r_2 \Delta b - \Delta d + \Delta g \frac{\hat{n}_1}{\delta_1 + w_2 \hat{n}_2} - \Delta b c_2 \hat{n}_2\right) n_3.$$

(Eq. S4)

The growth of species 3 is positive $(dn_3/dt > 0)$, that is, species 3 can invade successfully if $r_2 \Delta b - \Delta d + \Delta g \frac{\hat{n}_1}{\delta_1 + w_2 \hat{n}_2} - \Delta b c_2 \hat{n}_2 > 0$. Special cases, when one or two of the deltas are zero, render the analysis simpler. For example, if $\Delta g = \Delta b = 0$, and $\Delta d > 0$ (per capita birth rates and efficiency of using X_1 do not change, while withholding X_2 increases death rate since it needs extra energy to decompose within the cell), then species 3 cannot invade ($dN_3/dt < 0$ if $n_3 \approx 0$).

Now we consider the case when species 1 and 3 are present initially, and we are interested in condition of the invasion of species 2. The abundances of species 1 and 3 at the fixed point of this subsystem are denoted as $\hat{n}_1^*$ and $\hat{n}_3$, respectively. The method is similar to the one used above. The dynamics of n_2 in the linear approximation is:

$$\frac{dn_2}{dt} = \left(-r_2 \Delta b + \Delta d - \Delta g \frac{\hat{n}_1^*}{\delta_1 + w_2 \hat{n}_3} + \Delta b c_3 \hat{n}_3\right) n_2.$$

(Eq. S5)

In case of $\Delta g = \Delta b = 0$, and $\Delta d > 0$ that is when withholding X_2 increases death rate of species 3 comparing to species 2, then species 2 can invade in the species 1, 3 subsystem. That is, species 2 and 3 cannot coexist.

Another interesting case is when $\Delta g, \Delta d > 0$ at the same time. In this case, not secreting the byproduct X_2 means extra cost for the mutant species 3. However, it utilizes the byproduct X_1 of species 1 more effectively than species 2. According to (Eq. S4), species 3 can spread if:

$$-\Delta d + \Delta g \frac{\hat{n}_1}{\delta_1 + w_2 \hat{n}_2} > 0,$$

(Eq. S6)

and similarly, species 2 spreads in the species 1, 3 subsystem if:

$$\Delta d - \Delta g \frac{\hat{n}_1^*}{\delta_1 + w_2 \hat{n}_3} > 0.$$

(Eq. S7)

It is easy to show that $\hat{n}_1^* < \hat{n}_1$and assume that $\hat{n}_3 \geq \hat{n}_2$ (which, depending on the parameters can be valid or not), then the relations (Eq. S6) and (Eq. S7) can be satisfied simultaneously. In this case, species 2 and 3 mutually invade each other, thus all three species will be in coexistence. If only (Eq. S6) is true and (Eq. S7) is not, then the cheater mutant excludes the cooperator one. However, the evolutionary history depends on the trade-off between g and d. It is natural to assume that an increased efficiency in consumption entails an increase in death rate, so these two variables are in positive trade-off. This is a reasonable assumption, as the increased performance of any metabolic machinery of incurs an energetic cost that either decreases reproduction or growth rate or increases

the death rate. The evolution of these correlated traits via adaptive dynamics is discussed in SI 6 and SI 8.

In turn, we examine the invasion dynamics of a third species when there is self-inhibition of products ($h_i > 0, \forall i$). Since there is only one positive stable fixed point in the model of cross-feeding with self-inhibition (SI 5), we can use the same analysis as we did for the case without inhibition. Again, we consider the species 1, 2 subsystem. From the definition of fixed point, it follows that

$$r_2 b_2 - d_2 + g_2 \frac{k_1 \hat{n}_1}{\delta_1 + w_2 \hat{n}_2} - b_2 c_2 \hat{n}_2 - h_2 \frac{k_2 \hat{n}_2}{\delta_1 + w_1 \hat{n}_1} = 0,$$

where $\hat{n}_1$ and $\hat{n}_2$ are the positive fixed point of the dynamics. Using the similar notations as above and denote $h_3 = h_2 + \Delta h$, we receive that the in the linear limit the mutant species 3 dynamics is:

$$\frac{dn_3}{dt} = \left(r_2 \Delta b - \Delta d + \Delta g \frac{\hat{n}_1}{\delta_1 + w_2 \hat{n}_2} - \Delta b c_2 \hat{n}_2 - \Delta h \frac{k_2 \hat{n}_2}{\delta_1 + w_1 \hat{n}_1} \right) n_3.$$

So rare species 3 can invade successfully if $r_2 \Delta b - \Delta d + \Delta g \frac{\hat{n}_1}{\delta_1 + w_2 \hat{n}_2} - \Delta b c_2 \hat{n}_2 - \Delta h \frac{k_2 \hat{n}_2}{\delta_1 + w_1 \hat{n}_1} > 0$, and practically the whole analysis remains identical with that presented previously, if $\Delta h = 0$ ($h_3 = h_2$).

7. Adaptive dynamics for cross-feeding without inhibition

We assume the following trade-off between the efficiency of consumption of the byproduct (g) and mortality (d):

$$g_i(z_i) = \frac{1}{2}\left(1 + \tanh\left(\frac{z_i - \bar{z}}{\sigma}\right)\right),$$
$$d_i(z_i) = \eta \max(0, z_i).$$

(Eq. S8)

where σ is the steepness of the sigmoid transition, $\bar{z}$ is the expected mean of the trait value z, and η is a scaling factor for positive values of z.

We assume that the underlying trait, z, can have any value, even negative, but as z increases, both dependent traits g and d increase. For a visual explanation of the trade-off, see Figure S5.

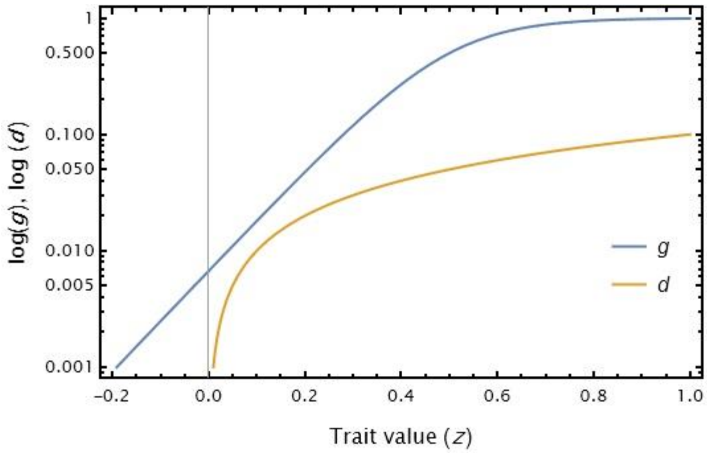

Figure S5. Relation of the trait value z to model parameters $\{g, d\}$. Note the logarithmic y scale. Parameters are $\bar{z} = 0.5, \sigma = 0.2, \eta = 0.1$.

The modified rate equations are:

$$\frac{\mathrm{d}n_1}{\mathrm{d}t} = n_1\left((b_1 r_1 - d_1) - b_1 c_1 n_1 + g_1 \frac{k_2 \sum_{j=2}^{a} n_j}{\delta_2 + w_1 n_1}\right),$$

$$\frac{\mathrm{d}n_i}{\mathrm{d}t} = n_i\left((b_i r_i - d_i) - b_i \sum_{l=1}^{a} c_l n_l + g_i \frac{k_1 n_1}{\delta_1 + w_2 \sum_{j=2}^{a} n_j}\right) \ (\forall i \geq 2),$$

where the growth of species 1 (the unchanging resident) is governed by the first equation, while the second equation describes the growth of any mutant of species 2 (itself included), and a is the total number of species in the system.

First, we examine the system when species 1 is not allowed to mutate, only species 2 (see Figure S6).

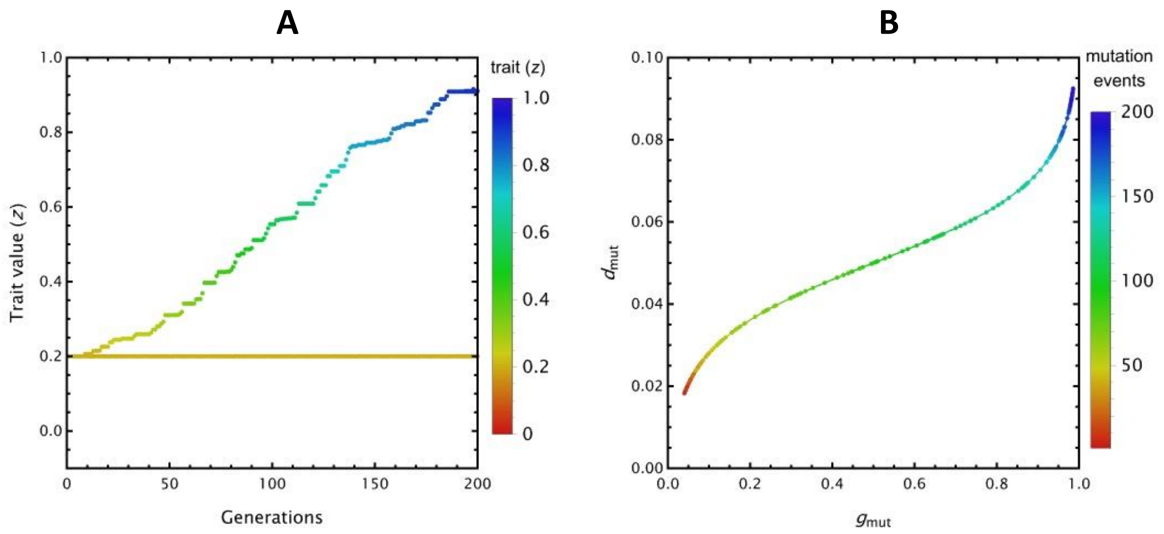

Figure S6. Adaptive dynamics within the model of cross-feeding without self-inhibition ($h_i = 0, \forall i$). A: Trait value against generations; colours correspond to trait values. B: Time evolution of the invading mutant's trait-dependent parameter values $\{g_{mut}, d_{mut}\}$; colours correspond to mutation events. Parameters are $\{t_{inv} = 10^4, n_{inv} = 0.01, \mu_{SD} = 10^{-2}, n_\theta = 10^{-3}, G = 200, z_1 = z_2 = 0.2, b_1 = b_2 = 1, c_1 = c_2 = 1, k_1 = k_2 = 1, r_1 = r_2 = 1, \delta_1 = \delta_2 = 0.1, w_1 = w_2 = 1, \bar{z} = 0.5, \sigma = 0.2, \eta = 0.1\}$.

Starting each simulation from a different initial trait value for species 2 shows the different evolutionary trajectories that lead to one of two equilibrium trait values (see Figure S7).

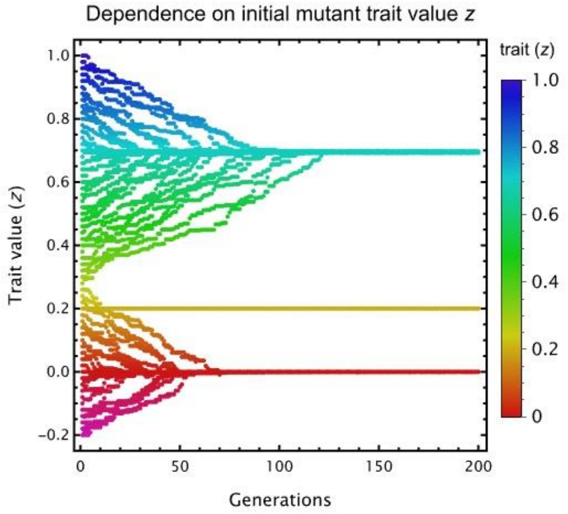

Figure S7. Dependence of adaptive dynamics outcome on initial mutant trait value z, iterated over the range $z_{mut\ 1} = \{-0.2, 1.0\}$*. Colour corresponds to trait value. Parameters are* $\{t_{inv} = 10^4, n_{inv} = 0.01, \mu_{SD} = 10^{-2}, n_\theta = 10^{-3}, G = 200, z_1 = 0.2, b_1 = 1, b_2 = 0.1, r_1 = r_2 = 1, c_1 = 1, c_2 = 0.1, \delta_1 = \delta_2 = 0.1, k_1 = 1, k_2 = 0.1, w_1 = w_2 = 1, \bar{z} = 0.5, \sigma = 0.2, \eta = 0.1\}$*. Note that parameters are not the same for static and mutant lineages. The static resident species (species 1) remains unchanged (yellow line at* $z = 0.2$*). Below a certain initial* z*, cheating mutants can invade only (settling at the equilibrium trait value* $\hat{z} = 0$*). If a mutation large enough pushes the mutant above the separatrix around* $z \approx 0.26$*, the positive equilibrium* z *becomes the attractor, settling at* $z^* \approx 0.7$*. Both equilibria are stable against invading mutants.*

We use the per capita growth rates of species 1 and 2 (Eq. S2) to check if at the equilibrium, no mutant of species 2 can invade the system. The invasion growth rate G_2^* of a mutant of species 2 with trait z_2^* is:

$$G_2^* = b_2 r_2 - d_2(z_2^*) + g_2(z_2^*) \frac{k_1 \hat{n}_1}{w_2 \hat{n}_2 + \delta_1} - b_2 c_2 \hat{n}_2.$$

At equilibrium, $\frac{\mathrm{d}n_i}{\mathrm{d}t} = 0$ and $\frac{\mathrm{d}G_i}{\mathrm{d}z_i} = 0$ for both species, with equilibrium value $n_i = \hat{n}_i$. Analysis reveals that the growth rate is always negative for a mutant when species 1 and 2 are in equilibrium (see Figure S8). Note that species 1 is not stable evolutionary in this case but is kept fixed.

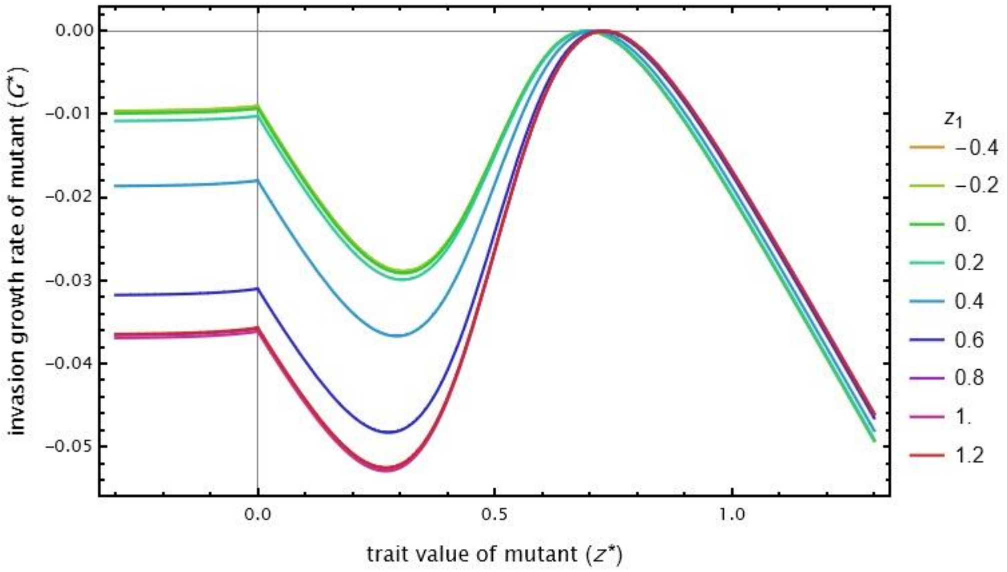

Figure S8. Invasion growth rate G_2^* of the mutant with trait z_2^* in equilibrium at different z_1 values (differently coloured curves). Note that the mutant growth rate is never positive, indicating that mutants cannot invade the system of species 1, 2 when they are in equilibrium. The root around $z = 0.7$ indicates the equilibrium value of z_2. Parameters are $\{b_1 = 1, b_2 = 0.1, r_1 = r_2 = 1, c_1 = 1, c_2 = 0.1, \delta_1 = \delta_2 = 0.1, k_1 = 1, k_2 = 0.1, w_1 = w_2 = 1, \bar{z} = 0.5, \sigma = 0.2, \eta = 0.1\}$.

In case both species can evolve, we define two general growth equations for the two mutant classes with respective abundances n_1 and n_2:

$$\frac{\mathrm{d}n_{1,i}}{\mathrm{d}t} = n_{1,i}\left(\left(b_1 r_1 - d(z_{1,i})\right) - b_1 c_1 \sum_j^{a_1} n_{1,j} + g(z_{1,i}) \frac{k_2 \sum_j^{a_2} n_{2,j}}{\delta_2 + w_1 \sum_j^{a_1} n_{1,j}}\right),$$

$$\frac{\mathrm{d}n_{2,i}}{\mathrm{d}t} = n_{2,i}\left(\left(b_2 r_2 - d(z_{2,i})\right) - b_2 c_2 \sum_j^{a_2} n_{2,j} + g(z_{2,i}) \frac{k_1 \sum_j^{a_1} n_{1,j}}{\delta_1 + w_2 \sum_j^{a_2} n_{2,j}}\right),$$

where a_i is the total number of species belonging to the ith mutant class. The two class of species, when their initial trait values allow, evolve towards better cross-feeders (see Figure S9).

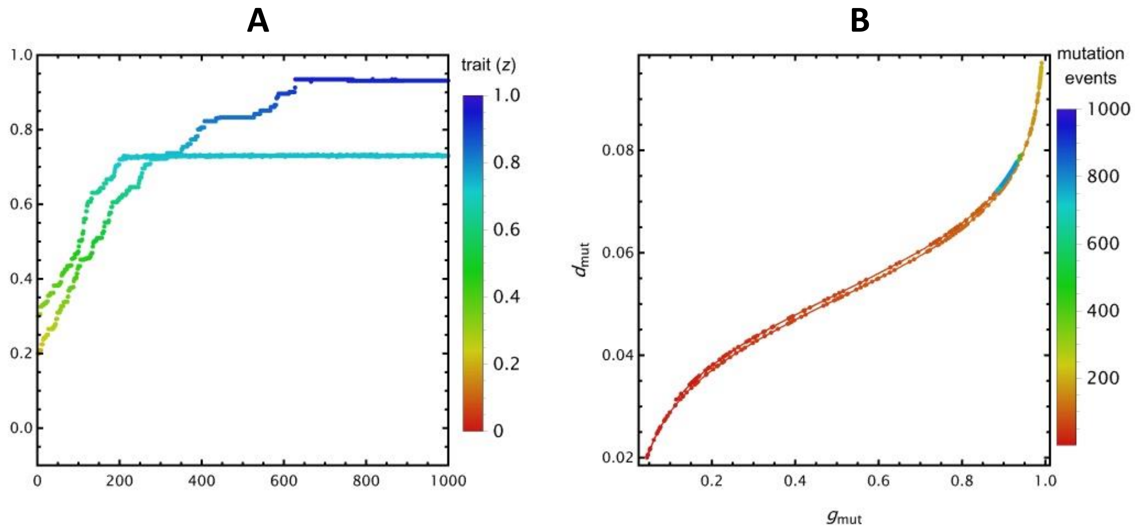

Figure S9. Adaptive dynamics of the cross-feeding model when both species can mutate and there is no inhibition. A: Trait value against generations; colours correspond to trait values. B: Time evolution of the invading mutant's trait-dependent parameter values $\{g_{mut}, d_{mut}\}$ for both classes (the trajectory of the second class is slightly

offset). Colours correspond to mutation events. Parameters are different for the two classes $\{t_{inv} = 5 \cdot 10^5, n_{inv} = 0.01, \mu_{SD} = 10^{-2}, n_\theta = 10^{-3}, G = 1000, z_1 = 0.2, z_2 = 0.3, b_1 = 1, b_2 = 0.1, c_1 = 1, c_2 = 0.1, k_1 = 1, k_2 = 0.1, r_1 = 1, r_2 = 1, \delta_1 = \delta_2 = 0.1, w_1 = 1, w_2 = 1, \bar{z} = 0.5, \sigma = 0.2, \eta = 0.1\}$. *While species of* N *have higher birth rates than species of* M*, they are present in less amounts at equilibrium (opacity of points correspond to relative equilibrium density). Nevertheless, they can evolve to better trait values than species of* M.

8. Adaptive dynamics for cross-feeding with inhibition

We take species 1 to be a static resident and only allow species 2 to mutate:

$$\frac{\mathrm{d}n_1}{\mathrm{d}t} = n_1\left((b_1 r_1 - d_1) - b_1 c_1 n_1 + g_1 \frac{k_2 \sum_{j=2}^{a} n_j}{\delta_2 + w_1 n_1} - h_1 \frac{k_1 n_1}{\delta_1 + w_2 \sum_{j=2}^{a} n_j}\right),$$

$$\frac{\mathrm{d}n_i}{\mathrm{d}t} = n_i\left((b_i r_i - d_i) - b_i \sum_{l=1}^{a} c_l n_l + g_i \frac{k_1 n_1}{\delta_1 + w_2 \sum_{j=2}^{a} n_j} - h_2 \frac{k_2 \sum_{j=2}^{a} n_j}{\delta_2 + w_1 n_1}\right) \quad (\forall i \geq 2),$$

where a is the total number of species. In case both species can evolve, we define two general growth equations for the two mutant classes with respective abundances n_1 and n_2:

$$\frac{\mathrm{d}n_{1,i}}{\mathrm{d}t} = n_{1,i}\left(\left(b_1 r_1 - d(z_{1,i})\right) - b_1 c_1 \sum_j^{a_1} n_{1,j} + g(z_{1,i}) \frac{k_2 \sum_j^{a_2} n_{2,j}}{\delta_2 + w_1 \sum_j^{a_1} n_{1,j}} - h_1 \frac{k_1 \sum_j^{a_1} n_{1,j}}{\delta_1 + w_2 \sum_j^{a_2} n_{2,j}}\right),$$

$$\frac{\mathrm{d}n_{2,i}}{\mathrm{d}t} = n_{2,i}\left(\left(b_2 r_2 - d(z_{2,i})\right) - b_2 c_2 \sum_j^{a_2} n_{2,j} + g(z_{2,i}) \frac{k_1 \sum_j^{a_1} n_{1,j}}{\delta_1 + w_2 \sum_j^{a_2} n_{2,j}} - h_2 \frac{k_2 \sum_j^{a_2} n_{2,j}}{\delta_2 + w_1 \sum_j^{a_1} n_{1,j}}\right),$$

where a_i is the total number of species belonging to the ith mutant class.

Simulations where only one or both species can evolve, starting from different initial mutant trait values are shown in Figure 4 in the main text.

9. Coexistence of species 1 and 2 for cross-facilitation

We study the case when only species 1 and species 2 are present (see Figure S10).

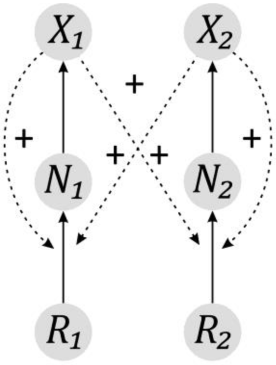

Figure S10. Model for cross-facilitation for two species. Dashed arrows indicate beneficial indirect effects, improving the resource consumption of all species N_i.

The dynamical equations of the concentrations can be estimated below and above by:

$$F_L(n_i) = n_\mathrm{i}((r_\mathrm{i} - c_i n_i) b_\mathrm{i} - d_\mathrm{i}) < \frac{\mathrm{d}n_\mathrm{i}}{\mathrm{d}t} < n_\mathrm{i}((r_\mathrm{i} - c_i n_i)(b_\mathrm{i} + s_{i1} + s_{i2}) - d_\mathrm{i}) = F_H(n_i),$$

for $i = 1,2$. If $r_i b_i - d_i > 0$, then $\frac{\mathrm{d}n_\mathrm{i}}{\mathrm{d}t} > 0$ for all $0 < n_i < n_{iL} = (r_i b_i - d_i)/(c_i b_i)$. That is, the densities of the species will increase if $0 < n_i < n_{iL}$. Contrarily, if $n_i > n_{iH} = \left(\frac{r_i((b_\mathrm{i}+s_{i1}+s_{i2})-d_i)}{(c_i(b_\mathrm{i}+s_{i1}+s_{i2})}\right) > 0$ then $\frac{\mathrm{d}n_\mathrm{i}}{\mathrm{d}t} < 0$, that is, densities will decrease if this condition is valid. Consequently, after a transient time, densities remain in a closed interval ($n_i \in [n_{iL}, n_{iH}]$) for all positive initial densities. This means that species 1 and species 2 coexist. Naturally, we have no information about the number of fixed points and their characteristics by this analysis.

10. Numerical investigation of stable fixed points for cross-facilitation

We examine the fixed point distribution for a large number of random parameter combinations for the equation system (Eq. 6) in the main text. For each combination, we perturb the parameter values $\{r_1 = r_2 = 1, \delta_1 = \delta_2 = 0.1, b_1 = b_2 = 1, c_1 = c_2 = 1, d_1 = d_2 = 0.01, k_1 = k_2 = 0.1, m_1 = m_2 = 1, s_{11} = s_{12} = s_{21} = s_{22} = 0.1\}$ independently by adding to each parameter a random value drawn from the normal distribution with mean 0 and standard deviation 0.5, ensuring that actual parameter values do not go below 10^{-3}. We calculate the numerical Jacobian of the system with the given parameters, substituting in the numerically calculated fixed points as the densities. For each fixed point, we check whether the eigen values of the Jacobian are all negatives. After extensive numerical testing (more than 100 000 independent random parameter combinations), we have not found any parameter combination which has more than 1 internal stable fixed point.

11. Existence of a single, globally stable internal fixed point for cross-facilitation

The per-capita growth rates of the subsystem of species 1 and 2 are:

$$G_1 = (r_1 - c_1 n_1)\left(b_1 + \frac{s_{11} k_1 n_1}{m_1 \delta_1 + k_1 n_1} + \frac{s_{12} k_2 n_2}{m_1 \delta_2 + k_2 n_2}\right) - d_1,$$

$$G_2 = (r_2 - c_2 n_2)\left(b_2 + \frac{s_{21} k_1 n_1}{m_2 \delta_1 + k_1 n_1} + \frac{s_{22} k_2 n_2}{m_2 \delta_2 + k_2 n_2}\right) - d_2.$$

At the internal fixed point, where $n_1, n_2 > 0$:

$$G_1 = G_2 = 0.$$

However, fixed points cannot be analytically calculated and simplified for this equation system.

Applying the Bendixson-Dulac theorem, where $h(n_1, n_2)$ is an arbitrary function, we may prove that there are no periodic solutions. Defining $h(n_1, n_2) = \frac{1}{n_1 n_2}$ and $f(n_1, n_2) = n_1', g(n_1, n_2) = n_2'$ yields:

$$\partial_{n_1}(hf) + \partial_{n_2}(hg) =$$

$$= -\frac{b_2 c_2 + c_2 s_{21} + c_2 s_{22}}{n_1} + \frac{c_2 m_2 s_{21} \delta_1}{n_1(m_2 \delta_1 + k_1 n_1)} - \frac{b_1 c_1}{n_2} + \frac{k_1 m_1 r_1 s_{11} \delta_1}{(m_1 \delta_1 + k_1 n_1)^2 n_2} - \frac{2 c_1 k_1 m_1 s_{11} \delta_1 n_1}{(m_1 \delta_1 + k_1 n_1)^2 n_2} -$$

$$- \frac{c_1 k_1^2 s_{11} n_1^2}{(m_1 \delta_1 + k_1 n_1)^2 n_2} - \frac{c_1 k_2 s_{12}}{m_1 \delta_2 + k_2 n_2} + \frac{k_2 m_2 r_2 s_{22} \delta_2}{n_1 (m_2 \delta_2 + k_2 n_2)^2} + \frac{c_2 m_2^2 s_{22} \delta_2^2}{n_1 (m_2 \delta_2 + k_2 n_2)^2},$$

which may be negative, depending on a complex combination that cannot be simplified (assuming $n_1, n_2 > 0$ and all parameters are positive).

While we did not manage to prove that only a single internal stable fixed point exists in this system, we have never found any other dynamical outcomes despite extensive numerical investigations (SI 10). We therefore conclude that even if multiple stable states are a possibility, they are highly unlikely to be realized and one can proceed as if the internal equilibrium point was unique. A characteristic phase plot is shown in Figure S11.

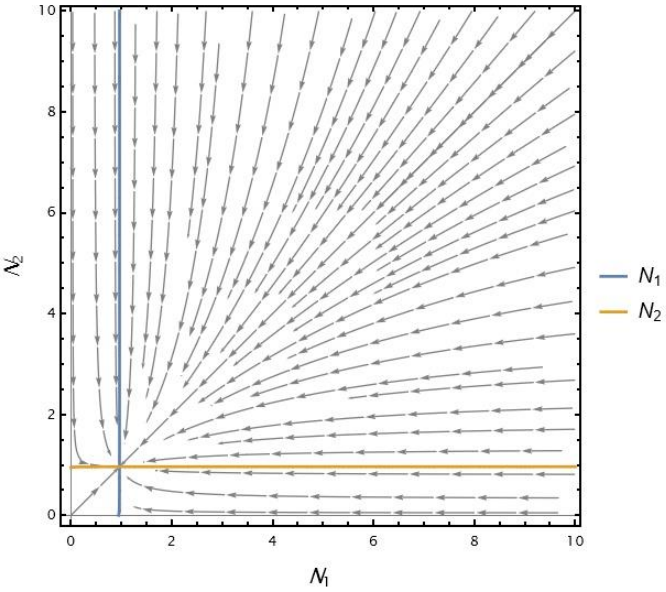

Figure S11. Phase plot of the cross-facilitation model. Parameters are: $\{b_1 = b_2 = 1, r_1 = r_2 = 1, d_1 = d_2 = 0.1, c_1 = c_2, \delta_1 = \delta_2 = 0.1, k_1 = k_2 = 1, w_1 = w_2 = 1, m_1 = m_2 = 1, s_{11} = s_{12} =, s_{21} = s_{22} = 1\}$.

12. Adaptive dynamics for cross-facilitation

We use the same trade-off functions as before (Eq. S8), now between the traits mortality (d) and production rate (k), as we assume that production is costly, and the higher the production rate, the higher the death rate is (see Figure S12):

$$k_i(z_i) = \frac{1}{2}\left(1 + \tanh\left(\frac{z_i - \bar{z}}{\sigma}\right)\right),$$
$$d_i(z_i) = \eta \max(0, z_i).$$

(Eq. S9)

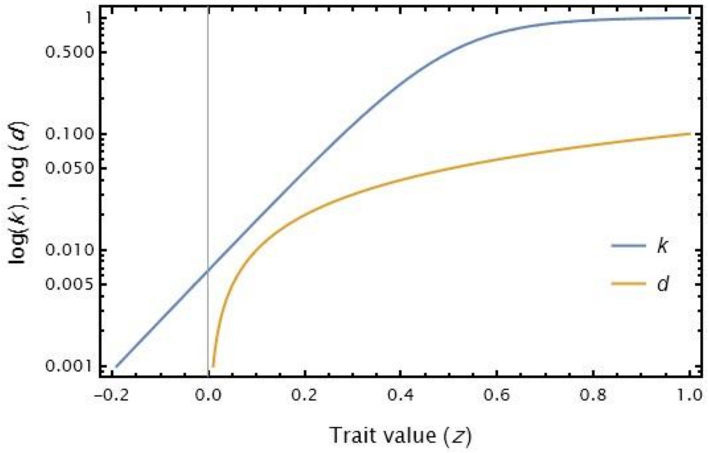

Figure S12. Relation of the trait value z to model parameters $\{k, d\}$*. Note the logarithmic y scale. Parameters are* $\bar{z} = 0.5, \sigma = 0.2, \eta = 0.1$.

Assuming that both species can evolve, we define two general growth equations for the two mutant classes with respective abundances n_1 and n_2, modifying the dynamical equations of (Eq. 6) in the main text:

$$\frac{\mathrm{d}n_{1,i}}{\mathrm{d}t} = n_{1,i}\left(\left(r_1 - c_1 \sum_j^{a_1} n_{1,j}\right)\left(b_1 + \frac{s_{11}\sum_j^{a_1} k(z_{1,j})n_{1,j}}{\delta_1 m_1 + \sum_j^{a_1} k(z_{1,j})n_{1,j}} + \frac{s_{12}\sum_j^{a_2} k(z_{2,j})n_{2,j}}{\delta_2 m_1 + \sum_j^{a_2} k(z_{2,j})n_{2,j}}\right) - d(z_{1,i})\right),$$

$$\frac{\mathrm{d}n_{2,i}}{\mathrm{d}t} = n_{2,i}\left(\left(r_2 - c_2\sum_{j}^{a_2} n_{2,j}\right)\left(b_2 + \frac{s_{21}\sum_j^{a_1} k(z_{1,j})n_{1,j}}{m_{21} + \sum_j^{a_1} k(z_{1,j})n_{1,j}} + \frac{s_{22}\sum_j^{a_2} k(z_{2,j})n_{2,j}}{m_{22} + \sum_j^{a_2} k(z_{2,j})n_{2,j}}\right) - d(z_{2,i})\right),$$

where a_i is the total number of species belonging to the ith mutant class.

Results for adaptive dynamics when both species can mutate are shown in Figure 5 in the main text. If z is close to zero (but not identical), several species can live together without exclusion (see Figure S13). This is because species with $z \leq 0$, their mortality is effectively zero ($d \approx 0$), and marginally small k (having no effect), which leads to a degenerate case where species can coexist in a neutral equilibrium.

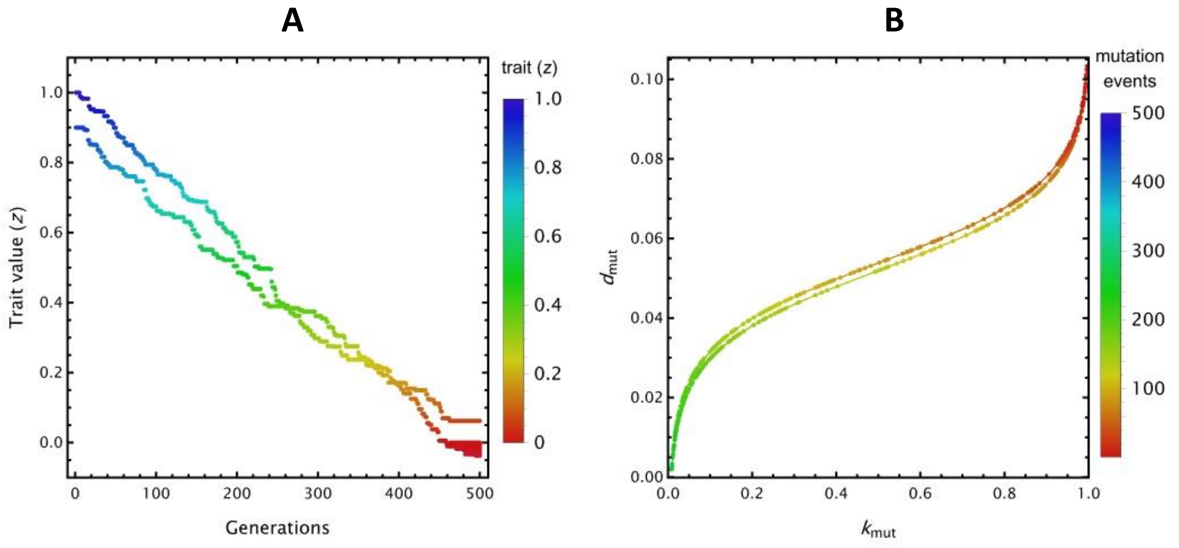

Figure S13. Adaptive dynamics of the cross-facilitation model when both species can mutate. A: Trait value against generations for both mutant classes; colours correspond to trait values, opacity to relative equilibrium density. B: Time evolution of the invading mutant's trait-dependent parameter values $\{k_{mut}, d_{mut}\}$ for both mutant classes (the trajectory of the second class is slightly offset); colours correspond to mutation events. Parameters are different for the two classes $\{t_{inv} = 5 \cdot 10^5, n_{inv} = 10^{-2}, \mu_{SD} = 10^{-2}, n_\theta = 10^{-3}, G = 550, z_1 = 1.0, z_2 = 0.9, b_1 = 1, b_2 = 0.1, c_1 = 1, c_2 = 0.1, r_1 = r_2 = 1, \delta_1 = \delta_2 = 0.1, m_1 = m_2 = 1, s_{11} = s_{12} = s_{21} = s_{22} = 0.1, \bar{z} = 0.5, \sigma = 0.2, \eta = 0.1\}$. Both classes of mutants evolve toward $z \approx 0$, where mortality d is minimal but production rate k is also reduced.

13. Adaptive dynamics for another model of cross-facilitation

In case of cross-facilitation of Figure 1B (and (Eq. 5) in the main text), products X_1 and X_2 have identical effects: they both improve the consumption of both resources R_1 and R_2. Accordingly, X_1 and X_2 can actually represent the same molecule, instead of two different products. Here, we modify the cross-facilitation case of (Eq. 5) to model the situation where product X_i improves the consumption of R_i only, but makes the resource available to any species. In this setup, we effectively differentiate between X_1 and X_2, as they cannot be the same molecule (see Figure S14).

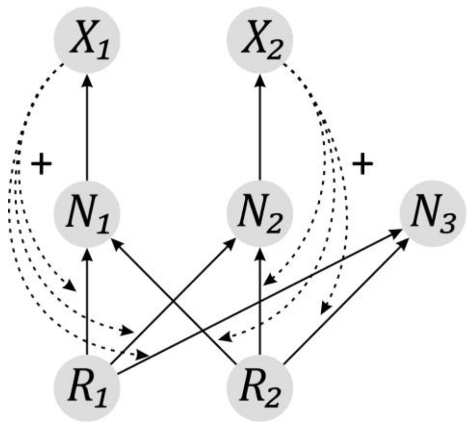

Figure S14. Model structure of a different type of cross-facilitation in which X_1 and X_2 have different metabolic roles, compared to Figure 1B in the main text, where they effectively represent the same molecule. N_3 is the non-producing mutant. Dashed arrows indicate catalytic aid over resource consumption for all species.

Ignoring species 3 for the cross-facilitation case, the dynamical equations are:

$$\frac{\mathrm{d}n_1}{\mathrm{d}t} = n_1\left(\rho_1 b_1 + \rho_1 \frac{s_{11}x_1}{m_1 + x_1} + \rho_2 \frac{s_{12}x_2}{m_1 + x_2} - d_1\right),$$

$$\frac{dn_2}{dt} = n_2\left(\rho_2 b_2 + \rho_2 \frac{s_{22}x_2}{m_2 + x_2} + \rho_1 \frac{s_{21}x_1}{m_2 + x_1} - d_2\right).$$

After substitution of fast resource and metabolite dynamics, equations for adaptive dynamics are:

$$\frac{\mathrm{d}n_{1,i}}{\mathrm{d}t} = n_{1,i}\left(\left(r_1 - c_1 \sum_j^{a_1} n_{1,j}\right)\left(r_1 b_1 + r_1 \frac{s_{11}\sum_j^{a_1} k(z_{1,j})n_{1,j}}{\delta_1 m_1 + \sum_j^{a_1} k(z_{1,j})n_{1,j}} + r_2 \frac{s_{12}\sum_j^{a_2} k(z_{2,j})n_{2,j}}{\delta_2 m_1 + \sum_j^{a_2} k(z_{2,j})n_{2,j}}\right) - d(z_{1,i})\right),$$

$$\frac{\mathrm{d}n_{2,i}}{\mathrm{d}t} = n_{2,i}\left(\left(r_2 - c_2 \sum_j^{a_2} n_{2,j}\right)\left(r_2 b_2 + r_1 \frac{s_{21}\sum_j^{a_1} k(z_{1,j})n_{1,j}}{\delta_1 m_2 + \sum_j^{a_1} k(z_{1,j})n_{1,j}} + r_2 \frac{s_{22}\sum_j^{a_2} k(z_{2,j})n_{2,j}}{\delta_2 m_2 + \sum_j^{a_2} k(z_{2,j})n_{2,j}}\right) - d(z_{2,i})\right).$$

When both species can mutate, results are qualitatively similar to that of the original cross-facilitation model (compare Figure S15 to Figure S13).

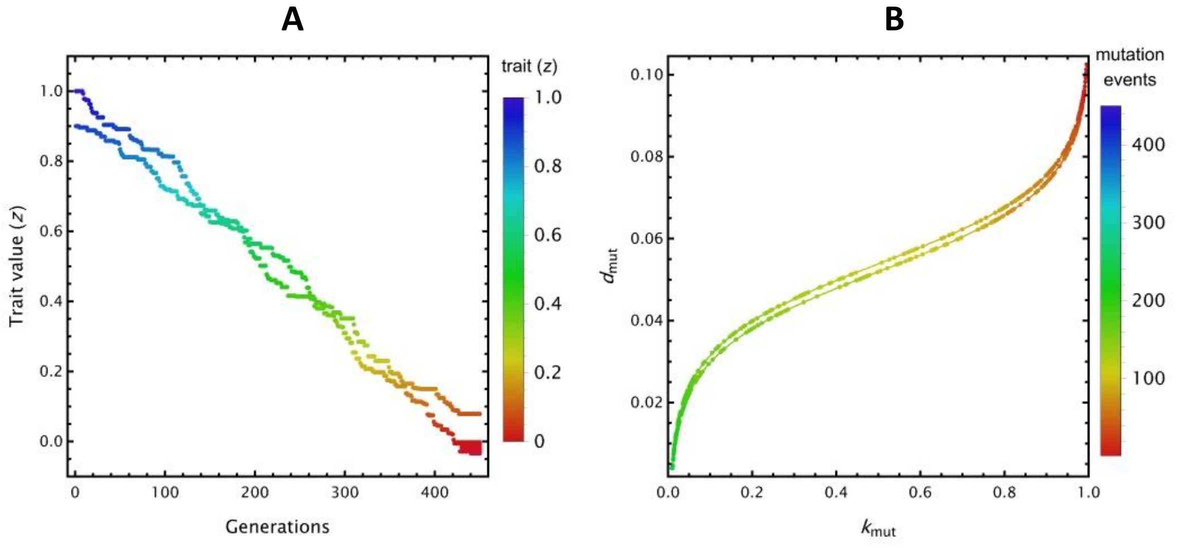

Figure S15. Adaptive dynamics of the cross-facilitation model when both species can mutate. A: Trait value against generations for both mutant classes; colours correspond to trait values, opacity to relative equilibrium density. B: Time evolution of the invading mutant's trait-dependent parameter values $\{k_{mut}, d_{mut}\}$ for both mutant classes (the trajectory of the second class is slightly offset); colours correspond to mutation events.

Parameters are different for the two mutant classes $\{n_{inv} = 0.01, t_{inv} = 5 \cdot 10^5, \mu_{SD} = 10^{-2}, n_\theta = 10^{-3}, G = 450, r_1 = r_2 = 1, \delta_1 = \delta_2 = 0.1, c_1 = 1, c_2 = 0.1, b_1 = 1, b_2 = 0.1, m_1 = m_2 = 1, s_{11} = s_{12} = s_{21} = s_{22} = 0.1, \bar{z} = 0.5, \sigma = 0.2, \eta = 0.1\}$.

14. Adaptive dynamics for a hybrid cross-feeding, cross-facilitation system

Next, we investigate the case where one of the species is a cross-feeder and the other is a cross-facilitator, as depicted in Figure 7.4 in the main text and detailed in Figure S16.

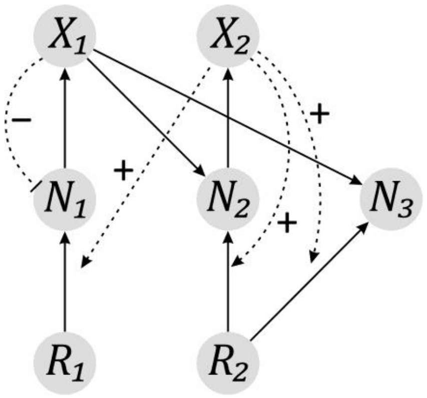

Figure S16. A possible hybrid case of syntrophy in which one species is a cross-feeder, the other is a cross-facilitator. Species N_3 is a mutant of the cross-facilitating species N_2 that does not produce anything but benefits from the catalytic effect of X_2 (dashed arrows).

Accordingly, species 1 enjoys a benefit of the enzyme of species 2, but plus the benefit of species 2 consuming its waste. On the other hand, species 2 enjoys the benefit of eating the waste of species 1 plus the benefit of its own external enzyme. The dynamical equations are as follows:

$$\frac{\mathrm{d}n_1}{\mathrm{d}t} = n_1\left(b_1\rho_1 + r_1\frac{s_{12}x_2}{m_1 + x_2} - h_1x_1 - d_1\right),$$
$$\frac{\mathrm{d}n_2}{\mathrm{d}t} = n_2\left(b_2\rho_2 + g_2x_1 + r_2\frac{s_{22}x_2}{m_2 + x_2} - h_2x_2 - d_2\right).$$

In turn, the metabolite dynamics read:

$$\frac{\mathrm{d}x_1}{\mathrm{d}t} = k_1n_1 - w_2n_2x_1 - \delta_1x_1,$$
$$\frac{\mathrm{d}x_2}{\mathrm{d}t} = k_2n_2 - \delta_2x_2.$$

We assume fast dynamics for the x_i by setting $\frac{\mathrm{d}x_i}{\mathrm{d}t} = 0$ to get the quasi-equilibrium equations:

$$x_1 = \frac{k_1n_1}{w_2n_2 + \delta_1},$$
$$x_2 = \frac{k_2n_2}{\delta_2}.$$

Substituting fast resource dynamics (of (Eq. 1) in the main text) and fast metabolite dynamics back into the dynamical equations yields:

$$\frac{dn_1}{dt} = n_1\left(b_1(r_1 - c_1 n_1) - d_1 - h_1\frac{k_1 n_1}{\delta_1 + w_2 n_2} + (r_1 - c_1 n_1)\frac{s_{12}k_2 n_2}{m_1\delta_2 + k_2 n_2}\right),$$
$$\frac{dn_2}{dt} = n_2\left(b_2(r_2 - c_2 n_2) - d_2 + g_2\frac{k_1 n_1}{\delta_1 + w_2 n_2} + (r_2 - c_2 n_2)\frac{s_{22}k_2 n_2}{m_2\delta_2 + k_2 n_2}\right).$$

Evolution mutates a unidimensional trait z in case of cross-facilitators. Their mortality d and enzyme production rate k depend directly on z through the same trade-off as before, as defined in (Eq. S9). However, in this setup, waste-consumption efficiency g_2 is also a property of cross-facilitators, and not of cross-feeders. We keep it as a static property. As a minor consequence, cross-feeders cannot consume the waste of other species hence they only have a single property d that could depend on a possible evolutionary trait for the cross-feeder class. For sake of simplicity, the cross-feeding species N_1 is kept as static and only the cross-facilitating species could mutate.

The dynamical equations prepared for adaptive dynamics are:

$$\frac{dn_{1,i}}{dt} = n_{1,i}\left(b_1\left(r_1 - c_1\sum_j^{a_1} n_{1,j}\right) - d_1 - h_1\frac{k_1\sum_j^{a_1} n_{1,j}}{\delta_1 + w_2\sum_j^{a_2} n_{2,j}} + \left(r_1 - c_1\sum_j^{a_1} n_{1,j}\right)\frac{s_{12}\sum_j^{a_2} k(z_{2,j})n_{2,j}}{m_1\delta_2 + \sum_j^{a_2} k(z_{2,j})n_{2,j}}\right),$$
$$\frac{dn_{2,i}}{dt} = n_{2,i}\left(b_2\left(r_2 - c_2\sum_j^{a_2} n_{2,j}\right) - d(z_i) + g_2\frac{k_1\sum_j^{a_1} n_{1,j}}{\delta_1 + w_2\sum_j^{a_2} n_{2,j}} + \left(r_2 - c_2\sum_j^{a_2} n_{2,j}\right)\frac{s_{22}\sum_j^{a_2} k(z_{2,j})n_{2,j}}{m_2\delta_2 + \sum_j^{a_2} k(z_{2,j})n_{2,j}}\right).$$

Result of a characteristic simulation is shown in Figure S17.

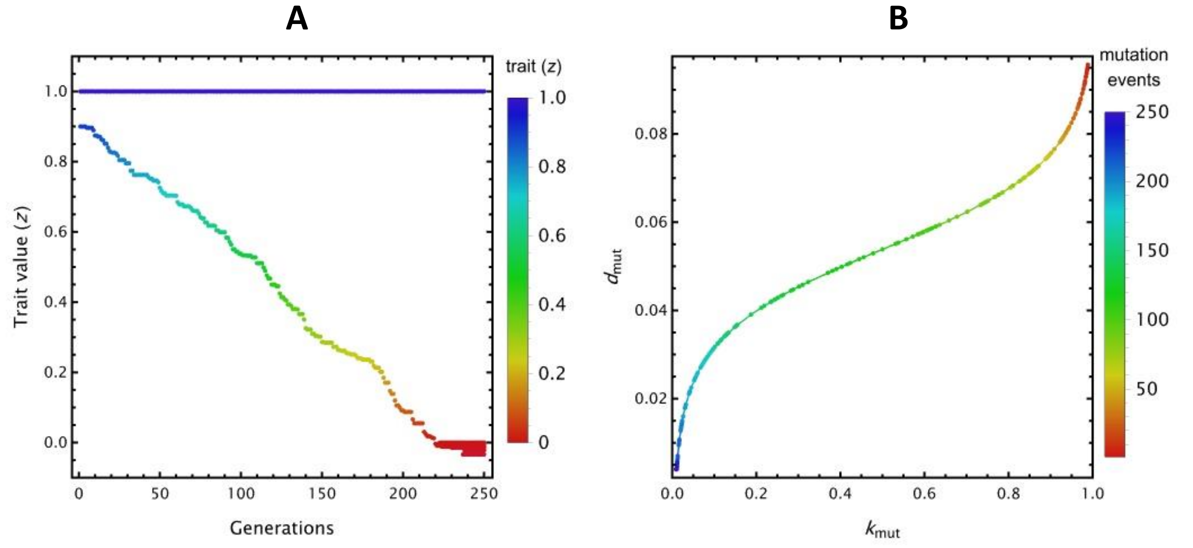

Figure S17. Adaptive dynamics of the hybrid cross-feeding and cross-facilitation model when only the cross-facilitating species can mutate. A: Trait value against generations for the cross-facilitating mutant classes (the blue line at $z_1 = 1$ indicates static cross-feeder species); colours correspond to trait values, opacity to relative equilibrium density. B: Time evolution of the invading mutant's trait-dependent parameter values $\{k_{mut}, d_{mut}\}$; colours correspond to mutation events. Parameters are $\{n_{inv} = 0.01, t_{inv} = 5\cdot 10^5, \mu_{SD} = 0.01, n_\theta = 10^{-3}, G = 250, r_1 = 1, r_2 = 1, \delta_1 = 1/10, \delta_2 = 1/10, c_1 = 1, c_2 = 0.1, b_1 = 1, b_2 = 0.1, m_1 = 1, m_2 = 1, d_1 = 0.01, k_1 = 1, h_1 = 1, g_2 = 1, w_2 = 1, s_{12} = s_{22} = 0.1, \bar{z} = 0.5, \sigma = 0.2, \eta = 0.1\}$.

15. Code to reproduce analyses and figures

Code files are supplied as separate Supplementary information files with this publication. The following Wolfram *Mathematica* notebooks are included.

**SI Fixed point analysis.nb**	Code to reproduce the fixed point analysis for cross-feeding (with or without inhibition) and cross-facilitation (main text Figures 2, 3 and SI 3-6, 9-11).
**SI Invasion fitness.nb**	Code to reproduce invasion fitness calculation for Figure S8.
**SI Adaptive dynamics.nb**	Code to reproduce adaptive dynamics analyses (main text Figures 4, 5, SI 7-8 and SI 12-14).

16. Supplementary references

1. Wintermute, E. H. & Silver, P. A. Emergent cooperation in microbial metabolism. Molecular Systems Biology 6, 407 (2010).
2. Mee, M. T., Collins, J. J., Church, G. M. & Wang, H. H. Syntrophic exchange in synthetic microbial communities. Proceedings of the National Academy of Sciences 111, E2149–E2156 (2014).
3. Pande, S. et al. Fitness and stability of obligate cross-feeding interactions that emerge upon gene loss in bacteria. The ISME Journal 8, 953–962 (2013).
4. Preussger, D., Giri, S., Muhsal, L. K., Oña, L. & Kost, C. Reciprocal fitness feedbacks promote the evolution of mutualistic cooperation. Current Biology 30, 3580–3590.e7 (2020).
5. Harcombe, W. Novel cooperation experimentally evolved between species. Evolution (2010).doi:10.1111/j.1558-5646.2010.00959.x
6. Wegener, G., Krukenberg, V., Riedel, D., Tegetmeyer, H. E. & Boetius, A. Intercellular wiring enables electron transfer between methanotrophic archaea and bacteria. Nature 526, 587–590 (2015).
7. Moissl-Eichinger, C. et al. Archaea are interactive components of complex microbiomes. Trends in Microbiology 26, 70–85 (2018).
8. Enke, T. N. et al. Modular assembly of polysaccharide-degrading marine microbial communities. Current Biology 29, 1528–1535.e6 (2019).
9. Datta, M. S., Sliwerska, E., Gore, J., Polz, M. F. & Cordero, O. X. Microbial interactions lead to rapid micro-scale successions on model marine particles. Nature Communications 7, (2016).
10. Rickard, A. H., Gilbert, P., High, N. J., Kolenbrander, P. E. & Handley, P. S. Bacterial coaggregation: an integral process in the development of multi-species biofilms. Trends in Microbiology 11, 94–100 (2003).
11. Kramer, J., Özkaya, Ö. & Kümmerli, R. Bacterial siderophores in community and host interactions. Nature Reviews Microbiology 18, 152–163 (2019).
12. Sorg, R. A. et al. Collective resistance in microbial communities by intracellular antibiotic deactivation. PLOS Biology 14, e2000631 (2016).
13. Yurtsev, E. A., Conwill, A. & Gore, J. Oscillatory dynamics in a bacterial cross-protection mutualism. Proceedings of the National Academy of Sciences 113, 6236–6241 (2016).
14. Vega, N. M. & Gore, J. Collective antibiotic resistance: mechanisms and implications. Current Opinion in Microbiology 21, 28–34 (2014).
15. Lee, H. H., Molla, M. N., Cantor, C. R. & Collins, J. J. Bacterial charity work leads to population-wide resistance. Nature 467, 82–85 (2010).
16. Gore, J., Youk, H. & Oudenaarden, A. van Snowdrift game dynamics and facultative cheating in yeast. Nature 459, 253–256 (2009).
17. Elias, S. & Banin, E. Multi-species biofilms: living with friendly neighbors. FEMS Microbiology Reviews 36, 990–1004 (2012).
18. Vidakovic, L., Singh, P. K., Hartmann, R., Nadell, C. D. & Drescher, K. Dynamic biofilm architecture confers individual and collective mechanisms of viral protection. Nature Microbiology 3, 26–31 (2017).
19. Lebeaux, D., Ghigo, J.-M. & Beloin, C. Biofilm-related infections: bridging the gap between clinical management and fundamental aspects of recalcitrance toward antibiotics. Microbiology and Molecular Biology Reviews 78, 510–543 (2014).
20. Jautzus, T., Gestel, J. van & Kovács, Á. T. Complex extracellular biology drives surface competition in lessigreaterBacillus subtilisless/igreater. (2022).doi:10.1101/2022.02.28.482363
21. West, S. A., Diggle, S. P., Buckling, A., Gardner, A. & Griffin, A. S. The social lives of microbes. Annual Review of Ecology, Evolution, and Systematics 38, 53–77 (2007).
22. Barrett, L. G., Bell, T., Dwyer, G. & Bergelson, J. Cheating, trade-offs and the evolution of aggressiveness in a natural pathogen population. Ecology Letters 14, 1149–1157 (2011).
23. Seth, E. C. & Taga, M. E. Nutrient cross-feeding in the microbial world. Frontiers in Microbiology 5, (2014).
24. Liu, Z. et al. Metabolite cross-feeding between Rhodococcus ruber YYL and Bacillus cereus MLY1 in the biodegradation of tetrahydrofuran under pH stress. Applied and Environmental Microbiology 85, (2019).
25. Kato, S., Haruta, S., Cui, Z. J., Ishii, M. & Igarashi, Y. Stable coexistence of five bacterial strains as a cellulose-degrading community. Applied and Environmental Microbiology 71, 7099–7106 (2005).
26. Flint, H. J., Bayer, E. A., Rincon, M. T., Lamed, R. & White, B. A. Polysaccharide utilization by gut bacteria: potential for new insights from genomic analysis. Nature Reviews Microbiology 6, 121–131 (2008).
27. Grondin, J. M., Tamura, K., Déjean, G., Abbott, D. W. & Brumer, H. Polysaccharide utilization loci: fueling microbial communities. Journal of Bacteriology 199, (2017).

28. Rakoff-Nahoum, S., Coyne, M. J. & Comstock, L. E. An ecological network of polysaccharide utilization among human intestinal symbionts. Current Biology 24, 40–49 (2014).
29. Rakoff-Nahoum, S., Foster, K. R. & Comstock, L. E. The evolution of cooperation within the gut microbiota. Nature 533, 255–259 (2016).
30. Porter, N. T. & Martens, E. C. Love thy neighbor: sharing and cooperativity in the gut microbiota. Cell Host & Microbe 19, 745–746 (2016).
31. Ross, B. D. et al. Human gut bacteria contain acquired interbacterial defence systems. Nature 575, 224–228 (2019).
32. Nogal, A., Valdes, A. M. & Menni, C. The role of short-chain fatty acids in the interplay between gut microbiota and diet in cardio-metabolic health. Gut Microbes 13, (2021).
33. Wang, C. et al. Protective effects of different Bacteroides vulgatus strains against lipopolysaccharide-induced acute intestinal injury, and their underlying functional genes. Journal of Advanced Research 36, 27–37 (2022).
34. Hammer, M. L. et al. The gut commensal Bacteroides vulgatus mpk reduces Candida albicans pathogenicity towards epithelial cells. Access Microbiology 3, (2021).
35. Lange, A. et al. Extensive mobilome-driven genome diversification in mouse gut-associatedlessigreaterbacteroides vulgatusless/igreatermpk. Genome Biology and Evolution 8, 1197–1207 (2016).
36. Mao, X., Stenuit, B., Polasko, A. & Alvarez-Cohen, L. Efficient metabolic exchange and electron transfer within a syntrophic trichloroethene-degrading coculture of dehalococcoides mccartyi 195 and syntrophomonas wolfei. Applied and Environmental Microbiology 81, 2015–2024 (2015).
37. Fröstl, J. M. & Overmann, J. Physiology and tactic response of the phototrophic consortium "Chlorochromatium aggregatum. Archives of Microbiology 169, 129–135 (1998).
38. Overmann, J. & Schubert, K. Phototrophic consortia: model systems for symbiotic interrelations between prokaryotes. Archives of Microbiology 177, 201–208 (2002).
39. Overmann, J. & Gemerden, H. van Microbial interactions involving sulfur bacteria: implications for the ecology and evolution of bacterial communities. FEMS Microbiology Reviews 24, 591–599 (2000).
40. Piccardi, P., Vessman, B. & Mitri, S. Toxicity drives facilitation between 4 bacterial species. Proceedings of the National Academy of Sciences 116, 15979–15984 (2019).
41. Zengler, K. & Zaramela, L. S. The social network of microorganisms - How auxotrophies shape complex communities. Nature Reviews Microbiology 16, 383–390 (2018).
42. Kuenen, J. G. Anammox bacteria: from discovery to application. Nature Reviews Microbiology 6, 320–326 (2008).
43. Lawson, C. E. et al. Metabolic network analysis reveals microbial community interactions in anammox granules. Nature Communications 8, (2017).
